# Zfand5 terminates TLR3/4 signaling and necroptosis by targeting TRIF to the proteasome for degradation

**DOI:** 10.1038/s41419-026-08946-0

**Published:** 2026-06-05

**Authors:** Wei-Ting Liao, Chia-Hsing Shen, Viriya Adhiguna Winarso, Yu-You Chen, Ke-Hsin Chen, Chun-Hsiang Chuang, En-Yi Liu, Miao-Hsien Wu, Chia-I Lien, Yu-Ching Chen, Ting-Yu Lai, Tsung-Hsien Chuang, Chih-Yuan Lee, Li-Chung Hsu

**Affiliations:** 1 Institute of Molecular Medicine, College of Medicine, National Taiwan University, Taipei, Taiwan, ROC; 2 Graduate Institute of Immunology, College of Medicine, National Taiwan University, Taipei, Taiwan, ROC; 3 Department of Surgery, National Taiwan University Hospital, Taipei, Taiwan, ROC; 4 Cardiovascular and Mitochondrial Related Disease Research Center, Hualien Tzu Chi Hospital, Buddhist Tzu Chi Medical Foundation, Hualien, Taiwan, ROC

**Keywords:** Toll-like receptors, Proteasome

## Abstract

Toll-like receptors (TLRs) are central to host defense and tissue repair, yet dysregulated TLR signaling contributes to inflammatory and autoimmune diseases. Although core TLR pathways have been defined, the mechanisms that terminate receptor signaling and restore immune homeostasis remain incompletely understood. Here, we identify the zinc finger protein Zfand5, a known proteasomal shuttling factor, as a selective negative regulator of TLR3- and TLR4-mediated inflammatory responses. Zfand5-deficient mice exhibit exacerbated sepsis and heightened cytokine production following TLR3/4 stimulation. Mechanistically, Zfand5 facilitates the proteasomal degradation of the adaptor protein TIR-domain-containing adapter-inducing interferon-β (TRIF) by bridging polyubiquitinated TRIF to the proteasome, thereby terminating downstream pro-inflammatory and type I interferon signaling. Loss of Zfand5 also enhances TRIF-dependent necroptosis upon TLR3/4 activation, further amplifying inflammation. These findings reveal an essential and previously unrecognized role for Zfand5 in regulating TRIF turnover and maintaining immune balance during innate immune responses.

## Introduction

The mammalian immune system comprises two major components: the innate and adaptive immune systems. The innate immune system serves as the first line of defense against invading pathogens. During infection, pattern recognition receptors (PRRs) on host cells recognize pathogen-associated molecular patterns (PAMPs) derived from these invaders [1]. Upon PAMP engagement, PRRs initiate signaling cascades that lead to the production of inflammatory cytokines, chemokines, and type I interferons (IFNs)[2]. Activated innate immune cells further potentiate and coordinate the adaptive immune responses, ultimately eliminating invading pathogens [3, 4]. In addition to PAMPs, endogenous molecules released from injured cells or stressed cells, termed danger-associated molecular patterns (DAMPs) and stress-associated molecular patterns (SAMPs), can also activate PRRs and provoke inflammation [5, 6]. Multiple classes of PRRs have been identified and characterized, including Toll-like receptors (TLRs), nucleotide-binding oligomerization domain (NOD)-like receptors (NLRs), C-type lectin-like receptors (CLRs), retinoic acid-inducible gene I (RIG-I)-like receptors (RLRs), and cytosolic DNA sensors such as cyclic GMP-AMP synthase (cGAS) and pyrin and HIN domain-containing (PYHIN) family proteins [2]. While the innate immune response is vital for pathogen clearance, tissue repair, and priming of adaptive immunity, its dysregulation can lead to harmful consequences, contributing to conditions such as sepsis, cancers, metabolic diseases, and autoinflammatory and autoimmune disorders [7]. Hence, tight regulation of the innate immune response is critical for maintaining immune homeostasis.

Among PRRs, TLRs are the most extensively studied. These receptors detect invading pathogens either at the cell surface or within intracellular endosomes [2, 8, 9]. To date, 13 TLR proteins have been identified in mammals: TLR1-10 in humans and TLR1-9 plus TLR11-13 in mice [10]. Based on subcellular localization, TLRs can be divided into two groups: those at the cell surface (TLR1, TLR2, TLR4, TLR5, and TLR6), which recognize microbial lipids, proteins, and lipoproteins; and those in endosomal membranes (TLR3, TLR7, TLR8, TLR9, and TLR11-13), which primarily detect nucleic acids from internalized pathogens [8]. TLRs initiate innate immune responses through activating downstream signaling upon engagement of the adaptor protein either Myeloid Differentiation Primary Response Gene-88 (MyD88) or Toll/IL-1 Receptor Domain-Containing Adaptor-Inducing IFN-β (TRIF), leading to the production of pro-inflammatory cytokines and type I IFNs [10–12]. With the exception of TLR3, all TLRs recruit MyD88 to trigger downstream signals. TLR4 is unique in utilizing both adaptors: upon recognition of bacterial lipopolysaccharide (LPS) at the plasma membrane, it signals via MyD88 with the aid of the sorting adaptor TIRAP/Mal, forming the Myddosome complex through recruitment of IRAK kinases [13]. This complex subsequently recruits and activates downstream signaling molecules, including TNF receptor-associated factor 6 (TRAF6) and transforming growth factor β-activated kinase 1 (TAK1), leading to the early activation of the IkappaB kinase (IKK)-nuclear factor kappa B (NF-κB) and mitogen-activated protein kinase (MAPK) pathways. Following this, TLR4 is internalized into endosomes, where it recruits TRIF via the sorting adaptor TRIF-related adaptor molecule (TRAM). This recruitment initiates the late-phase activation of IKK/NF-κB and MAPKs signaling and also triggers the activation of TRAF3 and TANK-binding kinase (TBK1), which in turn activates the transcription factor interferon regulatory factor 3 (IRF3)[14, 15]. While both MyD88- and TRIF-dependent pathways contribute to cytokine and chemokine production, TRIF is specifically required for type I IFN induction [16]. TLR3, which senses viral double-stranded RNA in endosomes, signals exclusively through TRIF to activate both the IKK-NF-κB and TBK1-IRF3 pathways [7].

To avoid detrimental inflammation and tissue damage, TLR signaling is tightly controlled by multiple negative regulatory mechanisms [17, 18]. These regulatory mechanisms operate at various levels, including receptor downregulation [19, 20], soluble/decoy receptor expression [21, 22], destabilization of signaling molecules [23], attenuation via post-translational modifications (PTMs)[24–26], endocytosis/lysosomal degradation [19], and feedback inhibition [18]. The adaptor proteins MyD88 and TRIF are central nodes in these pathways and are subject to post-transcriptional and post-translational regulation [13, 27–29]. For instance, MyD88 is regulated post-transcriptionally by non-coding RNAs such as miR155-5p [30] and miR-214 [31], which reduce its expression to limit inflammation. At the post-translational level, MyD88 is targeted for degradation by the E3 ubiquitin ligase casitas B-lineage lymphoma proto-oncogene-b (Cbl-b) [23]. Similarly, TRIF is targeted post-transcriptionally by non-coding RNAs, including miR181b-2 and miR-21-1 [32], and post-translationally by several E3 ubiquitin ligases, such as Cbl-b [23], TRAF3 [33], WW domain-containing protein 2 (WWP2) [34], and tripartite motif-containing protein 38 (TRIM38) [34, 35]. These mechanisms collectively ensure the proper control of TLR signaling and immune responses. Despite these insights, the detailed mechanisms and physiological consequences of TRIF and MyD88 regulation remain incompletely understood.

Zinc Finger AN1-Type Domain 5 (Zfand5) is a zinc finger-containing protein previously described as an atrogene associated with muscle atrophy [36], and is known to promote proteasomal degradation of ubiquitinated proteins, particularly under stress conditions [37, 38]. It has been implicated in muscle atrophy [36] and cancer [39, 40]. Earlier studies have suggested that Zfand5 may inhibit NF-κB activity [41]; however, these conclusions were primarily based on overexpression systems, and its endogenous function as well as physiological relevance remain poorly understood. Additionally, Zfand5 has been shown to interact with key innate immune signaling molecules, including IKKγ, TRAF6, and RIP proteins[41], yet the functional implications of these interactions have not been elucidated. Recent reports indicate that Zfand5 acts as a proteasome shuttling factor, binding polyubiquitinated substrates via its N-terminal A20-like zinc-finger domain and delivering them to the proteasome for degradation [36]. Additionally, its C-terminal domain (CTR) is both necessary and sufficient for proteasome activation, indicating that Zfand5 also functions as a proteasome activator [37, 38].

In this study, we identify Zfand5 as a novel negative regulator of TRIF-dependent TLR signaling. We demonstrate that Zfand5 interacts with TRIF and promotes its proteasomal degradation following TLR3/4 activation in macrophages. Loss of Zfand5 enhances activation of the IKK-NF-κB and TBK1-IRF3 pathways, leading to increased production of pro-inflammatory cytokines and type I IFNs. Moreover, Zfand5-deficient mice exhibit heightened inflammatory responses and increased susceptibility to LPS- and poly(I: C)-induced sepsis. These findings establish Zfand5 as a critical negative regulator of TLR3/4-mediated inflammation and reveal a previously unrecognized role for Zfand5 in maintaining innate immune homeostasis. Given the central role of TRIF signaling in type I interferon production, therapeutic targeting of Zfand5 may offer a promising strategy to boost antiviral immunity and improve vaccine efficacy.

## Results

### Zfand5 negatively regulates TLR3/4-driven innate immune responses

During microbial infection, host cells detect pathogens by recognizing PAMPs through various PRRs, thereby initiating inflammatory responses to eliminate the invaders [2]. Given that PRR-driven innate immune response must be tightly regulated to ensure proper initiation and resolution of inflammation [42], we investigated how macrophages respond to the TLR4 agonist lipopolysaccharide (LPS) by analyzing publicly available microarray data from the National Center for Biotechnology Information (NCBI). Through this analysis, we identified a zinc finger-containing protein, Zfand5, that was upregulated in macrophages following LPS stimulation. To explore the function of Zfand5 during inflammation, we employed a CRISPR/Cas9-mediated editing system to generate *Zfand5*^–/–^ RAW 264.7 macrophages. We then assessed the impact of Zfand5 deletion on TLR-driven innate immune responses in these macrophages after stimulation of various TLR agonists. Upon stimulation with TLR3 agonist poly(I: C) or TLR4 agonist LPS, Zfand5-deficient RAW 264.7 macrophages showed significantly increased mRNA levels of inflammatory genes, including *Il1b*, *Il6*, *Tnf*, *Ifnb*, and *Rantes*, compared to wild-type cells (Supplementary Fig. 1a). In contrast, stimulation with Pam_3_CSK_4_ and Zymosan (TLR2 agonists), R837 (TLR7 agonist), or CpG-DNA (TLR9 agonist) led to only minor differences in cytokine and IFN gene expression between wild-type and Zfand5-depleted cells (Supplementary Fig. 1a, b). Consistent with these results, the activation of IKK, TBK, and IRF3 was enhanced in Zfand5-deficient RAW 264.7 cells following LPS and poly(I:C) stimulation (Supplementary Fig. 1c, d), whereas TLR2-driven signaling remained comparable between control and Zfand5-deleted cells (Supplementary Fig. 1e). We further examined the nuclear translocation of NF-κB/p65 and IRF3, two key transcription factors critical for the induction of inflammatory cytokines and type I IFNs. Our results showed that Zfand5 deficiency significantly promoted NF-κB/p65 and IRF3 translocation into the nucleus after LPS or poly(I:C) stimulation (Supplementary Fig. 1f–i). In contrast, Pam_3_CSK_4_-induced NF-κB/p65 nuclear translocation was similar in wild-type and Zfand5-depleted cells (Supplementary Fig. 1j). Taken together, these results indicate that Zfand5 selectively regulates TLR3/4-activated signaling pathways, but not those mediated by TLR2, TLR7, or TLR9.

**Fig. 1:**
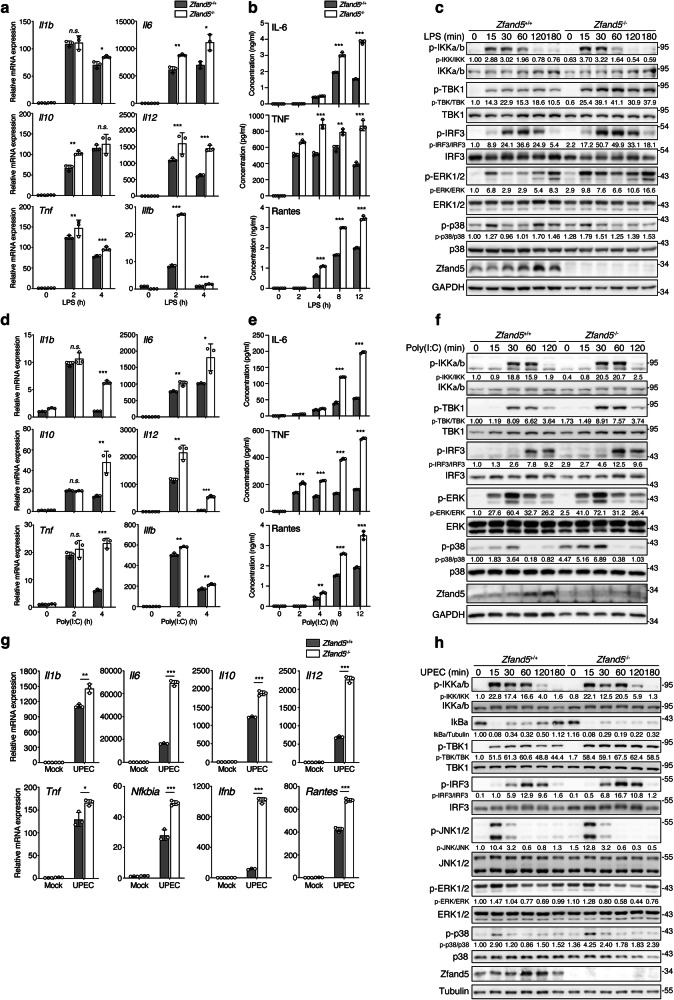
Zfand5 negatively regulates TLR3/4-triggered innate immune responses. BMDMs from wild-type (*Zfand5*^+/+^) or *Zfand5*^–/–^ mice were treated with LPS (100 ng/ml) (**a–c**) or poly(I:C) (30 μg/ml) (**d–f**) for the indicated times. The mRNA expression of the indicated genes was analyzed by RT-qPCR (**a, d**). Cytokine concentrations in culture supernatants were measured by ELISA (**b, e**). Phosphorylation of IKKα/β, TBK1, IRF3, and MAPKs, along with total protein levels, was assessed by immunoblotting (**c, f**). BMDMs from *Zfand5*^+/+^ or *Zfand5*^–/–^ mice were infected with UPEC for the indicated times. The expression levels of the indicated mRNAs were analyzed by RT-qPCR (**g**). Phosphorylation of signaling proteins and expression levels of the indicated proteins were analyzed by immunoblotting (**h**). Band intensities are quantified and presented as fold changes relative to untreated controls after normalization to their unphosphorylated forms, and are shown below. Statistical significance was determined using Student’s *t* test (*P < 0.05, **P < 0.01, ***P < 0.001; ns not significant). Data are representative of three independent experiments performed in triplicate and are presented as mean ± SD (error bars).

To investigate the physiological function of Zfand5, we generated *Zfand5*^F/F^ (*loxP*-flanked *Zfand5*) mice by inserting two *loxP* sites into the third and fifth introns of the *Zfand5* gene through two rounds of homologous recombination. For systemic deletion, *Zfand5*^F/F^ mice were crossed with transgenic mice expressing Cre recombinase under the control of the SRY-box containing gene 2 (Sox2) promoter [43] (Supplementary Fig. 2a). A male *Zfand5*^F/+^:Sox2-Cre mouse was then mated with a *Zfand5*^F/F^ female to generate *Zfand5*^F/-^ offspring, which were backcrossed with C57BL/6 J mice to obtain *Zfand5*^+/-^ mice. Subsequent intercrossing of *Zfand5*^+/-^ mice produced offspring at expected Mendelian ratios (Supplementary Fig. 2b), indicating that Zfand5 is dispensable for mouse embryonic development. Both *Zfand5*^+/+^ and *Zfand5*^–/–^ mice exhibited comparable body weights (Supplementary Fig. 2c). Peripheral blood counts were also similar between *Zfand5*^+/+^ and *Zfand5*^–/–^ mice (Supplementary Table 1). Bone marrow cells from *Zfand5*^+/+^ and *Zfand5*^–/–^ mice yielded similar numbers of bone marrow-derived macrophages (BMDMs) upon differentiation (Supplementary Fig. 2d, e). To assess the effect of Zfand5 on TLR-driven inflammatory responses in primary macrophages, we stimulated *Zfand5*^+/+^ and *Zfand5*^–/–^ BMDMs with various TLR agonists. Consistent with the results from RAW 264.7 macrophages, stimulation with poly(I:C) and LPS led to significantly elevated mRNA levels of inflammatory genes, including *Il1b*, *Il6*, *Il10*, *Il12*, *Tnf*, and *Ifnb*, in *Zfand5*^–/–^ BMDMs (Fig. 1a–d). In line with these transcriptional changes, *Zfand5*-deficient BMDMs produced higher levels of cytokines, including IL-6, TNF, and Rantes (Fig. 1b, e), and exhibited enhanced activation of IKK, TBK1, IRF3, and MAPKs following treatment with LPS and poly(I:C) (Fig. 1c, f). By contrast, TLR2 signaling in response to Pam_3_CSK_4_ was largely unaffected by Zfand5 depletion, despite a slight increase in the mRNA and protein levels of a few inflammatory genes (Supplementary Fig. 3). Furthermore, Zfand5 also regulated TLR3/4-induced immune responses in bone marrow-derived dendritic cells (BMDCs) (Supplementary Fig. 4), indicating that its regulatory role is not restricted to macrophages.

To further evaluate the role of Zfand5 in antimicrobial defense, we infected *Zfand5*^+/+^ and *Zfand5*^–/–^ BMDMs with uropathogenic *Escherichia coli* (UPEC), a pathogen known to activate TLR4 signaling [44]. In *Zfand5*^–/–^ BMDMs, UPEC infection significantly elevated the mRNA expression of cytokines and type I IFNs, including *Il1b*, *Il6*, *Il10*, *Il12*, *Tnf*, *Nfkbia*, *Ifnb*, and *Rantes*, compared to wild-type BMDMs (Fig. 1g). This was accompanied by enhanced activation of IKK, TBK1, IRF3, and MAPKs (Fig. 1h). Collectively, these findings suggest Zfand5 as a negative regulator of TLR3/4-driven immune responses, playing a critical role in dampening inflammation and maintaining immune homeostasis.

### Loss of Zfand5 aggravates LPS- and poly(I:C)-induced septic shock

**Fig. 2:**
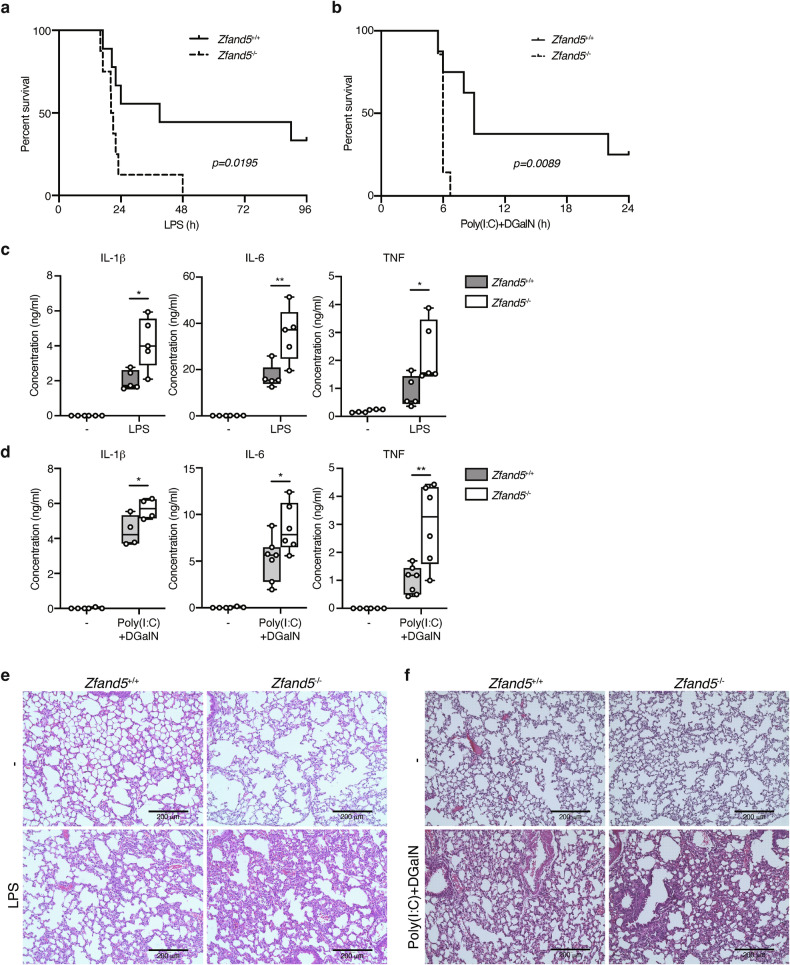
Depletion of Zfand5 exacerbates LPS- and poly(I:C)-induced septic shock in mice. Survival curves of *Zfand5*^+/+^ (solid line, *n* = 9) and *Zfand5*^–/–^ mice (dotted line, *n* = 8) following intraperitoneal injection of either LPS (15 mg/kg body weight) (**a**) or poly(I:C) (2 mg/kg) combined with D-galactosamine (D-GalN, 0.5 mg/g) (**b**). Survival differences were analyzed using the log-rank (Mantel-Cox) test. Serum cytokine levels measured by ELISA in *Zfand5*^+/+^ and *Zfand5*^–/–^ mice 4 hours after LPS (**c**) or poly(I:C) plus D-GalN (**d**) injection. Haematoxylin and eosin (H&E) staining of lung tissue sections collected 4 h post-injection from *Zfand5*^+/+^ and *Zfand5*^–/–^ mice treated with LPS (**e**) or poly(I:C) plus D-GalN (**f**) (lower panels), and from PBS-injected controls (upper panels). Objective magnification, 20×. Scale bar, 200 μm. Statistical significance was determined using Student’s *t* test (*P < 0.05, **P < 0.01, and ***P < 0.001). Data are presented as mean ± SD (Error bars).

Sepsis is predominantly an inflammatory disorder driven by excessive activation of the innate immunity [45]. To determine whether Zfand5 modulates TLR3/4-mediated immune responses in vivo, we intraperitoneally administered a lethal dose of LPS or poly(I:C) in combination with D-galactosamine (D-GalN) to *Zfand5*^+/+^ and *Zfand5*^–/–^ mice. Following LPS challenge, *Zfand5*^–/–^ mice exhibited significantly higher mortality (100% vs. 66%) within 96 h compared to wild-type controls (Fig. 2a). A similar pattern was observed in response to poly(I:C)/D-GalN, where *Zfans5*^–/–^ mice were more susceptible to death (100% vs. 75%) within 24 hours (Fig. 2b). These survival differences were accompanied by markedly elevated serum levels of pro-inflammatory cytokines, including IL-6, IL-1β, and TNF, in *Zfand5*^–/–^ mice following LPS or poly(I:C) challenge (Fig. 2c, d), suggesting a hyperinflammatory response in the absence of Zfand5. Histological examination further supported these findings, showing increased immune cell infiltration in lung tissues of *Zfand5*^–/–^ mice compared to wild-type controls after both LPS and poly(I:C)/D-GalN administration (Fig. 2e, f). Collectively, these results demonstrate that Zfand5 acts as a negative regulator of TLR3/4-induced pro-inflammatory cytokine production, and its loss exacerbates the severity and mortality of septic shock.

### Zfand6 modulates TLR4-triggered inflammatory response through a mechanism distinct from Zfand5

Zfand6 shares close structural homology with Zfand5 [46]. To investigate whether Zfand6 also regulates TLR4-induced immune response, we knocked down *Zfand6* expression in RAW 264.7 cells using lentivirus-mediated shRNA, followed by stimulation with LPS. Upon LPS treatment, Zfand6 depletion led to significant increase in *Il6* mRNA expression and a decrease in *Il10*, whereas the mRNA levels of *Il1b*, *Il12*, *Tnf*, *Ifnb*, and *Rantes* remained largely unchanged (Supplementary Fig. 5a). Importantly, *Zfand6* knockdown did not alter *Zfand5* mRNA expression (Supplementary Fig. 5b). Furthermore, TLR4 signaling, monitored via the activation of IKK, TBK1, MAPKs, and IRF3, was comparable between Zfand6-depleted and control RAW 264.7 macrophages (Supplementary Fig. 5c). These data suggest that Zfand6 has only a modest effect on TLR4 signaling and likely modulates TLR4-mediated immune responses through a mechanism distinct from that of Zfand5.

### Zfand5 facilitates proteasome-dependent TRIF degradation following TLR3/4 activation

**Fig. 3:**
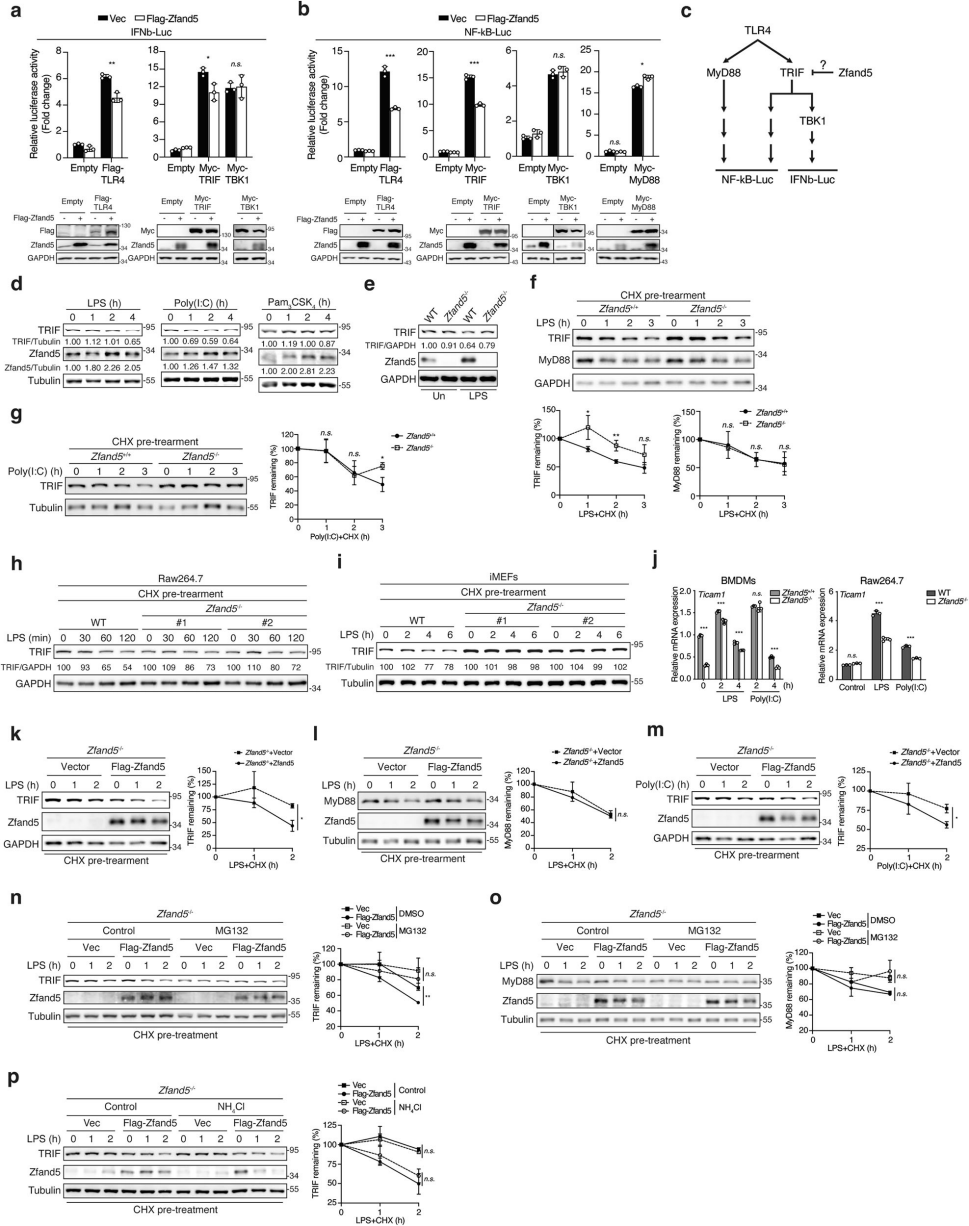
TLR3/4 activation-induced TRIF degradation is attenuated in Zfand5-depleted macrophages. HEK293T cells were co-transfected with IFNβ-Luc (**a**) or NF-κB-Luc (**b**) reporter constructs along with Flag-tagged Zfand5 and Myc-tagged TRIF, TBK1, or MyD88 as indicated. Cells were harvested after 36 hours, and luciferase activity was analyzed using the dual-luciferase reporter assay. The expression of transfected proteins in cell lysates was confirmed by immunoblotting (lower panels). **c** Schematic diagram illustrating the proposed role of Zfand5 in TLR3/4-mediated signaling. **d** BMDMs were stimulated with LPS (100 ng/ml), poly(I:C) (30 μg/ml), or Pam_3_CSK_4_ (100 ng/ml) for the indicated times. **e** *Zfand5*^+/+^ and *Zfand5*^–/–^ RAW 264.7 cells were treated with LPS (100 ng/ml) for 4 h. The protein levels of TRIF, MyD88, Zfand5, and tubulin in cell lysates were analyzed by immunoblotting. Band intensities are presented as fold changes relative to untreated control cells after normalization to Tubulin expression, and are shown below. *Zfand5*^+/+^ and *Zfand5*^–/–^ BMDMs were pretreated with cycloheximide (CHX, 2 μg/ml) for 30 mins and then stimulated with LPS (100 ng/ml) (**f**) or poly(I:C) (30 μg/ml) (**g**) for the indicated times. Cell lysates were analyzed by immunoblotting using the indicated antibodies. TRIF and MyD88 band intensities are normalized to GAPDH or tubulin expression and compared to untreated cells. Quantification of three independent experiments is shown in the right or bottom panels. **h, i** Wild-type (WT) and *Zfand5*^–/–^ RAW 264.7 cells or iMEFs were pretreated with CHX (2 μg/ml) for 30 mins and stimulated with LPS (100 ng/ml) for the indicated times. TRIF levels were analyzed as in (**f, g**). **j** WT and *Zfand5*^–/–^ BMDMs or RAW 264.7 cells were treated with LPS (100 ng/ml) or poly(I:C) (30 μg/ml) for the indicated times. The expression levels of the indicated mRNA were analyzed by RT-qPCR. **k–p** *Zfand5*^–/–^ RAW 264.7 cells were reconstituted with a Tet-inducible vector or Flag-tagged Zfand5 construct. **k–m** Reconstituted cells were pretreated with CHX (2 μg/ml) for 30 mins, followed by stimulation with LPS (100 ng/ml) or poly(I:C) (100 μg/ml) for the indicated times. Cells were pretreated with CHX (2 μg/ml) alone or CHX plus MG132 (2 μM) (**n, o**) or NH_4_Cl (2 μM) (**f**) for 30 mins, followed by stimulation with LPS (100 ng/ml) and CHX in the presence or absence of MG132 (**n, o**) or NH_4_Cl (**p**) for the indicated times. Cell lysates were analyzed by immunoblotting with the indicated antibodies. TRIF band intensities were normalized to GAPDH or tubulin expression and compared with untreated cells. Quantification of three independent experiments is shown in the right panels. Statistical significance was determined using Student’s *t* test (*P < 0.05, **P < 0.01, ***P < 0.001; ns, not significant). Data are representative of three independent experiments performed in triplicate and are presented as mean ± SD (error bars).

We next investigated how Zfand5 regulates TLR3/4-mediated signaling. Using luciferase reporter assays, we found that Zfand5 suppressed NF-κB and IFNβ promoter activities when TLR4 or TRIF, but not TBK1, was co-expressed in HEK293T cells. In addition, Zfand5 did not influence MyD88-mediated NF-κB activation (Fig. 3a, b). These findings suggest that Zfand5 specifically modulates TLR3/4 signaling by targeting TRIF (Fig. 3c). Zfand5 has been characterized as both a proteasome shuttling factor and a proteasome activator [37, 38]. Consistent with a previous report [23], we observed a reduction in TRIF protein levels following LPS stimulation (Fig. 3d). In addition to LPS, our results also revealed that poly(I:C), but not Pam_3_CSK_4_, induced a similar TRIF reduction, whereas Zfand5 protein levels were elevated in response to all three stimuli (Fig. 3d). Notably, LPS-induced degradation of TRIF, but not MyD88, was observed in wild-type RAW 264.7 cells but was absent in *Zfand5*^–/–^ RAW 264.7 cells (Fig. 3e), implicating Zfand5 in the regulation of TRIF turnover. To directly assess TRIF protein stability, we treated cells with cycloheximide (CHX) to inhibit de novo protein synthesis and then stimulated them with LPS or poly(I:C). In Zfand5-deficient cells, TRIF degradation was impaired in response to both stimuli (Fig. 3f–h), while MyD88 protein levels remained unaffected, confirming that Zfand5 specifically controls TRIF protein stability. Zfand5-dependent TRIF degradation was also observed in immortalized mouse embryonic fibroblasts (iMEFs) (Fig. 3i), indicating that this regulatory mechanism is not restricted to immune cells. Additionally, a slight reduction in *Trif* mRNA was observed in both BMDMs and RAW 264.7 cells lacking Zfand5 upon LPS and poly(I:C) stimulation, but this effect was modest (Fig. 3j), suggesting that Zfand5 primarily regulates TRIF at the post-translational level. Supporting the specificity of this mechanism, Zfand6 depletion did not affect the degradation of TRIF or MyD88 upon LPS stimulation (Supplementary Fig. 5d, e). To further determine whether Zfand5 regulates TLR3/4-driven immune responses through TRIF, we generated *Zfand5*^–/–^*Trif*^–/–^ immortalized BMDMs and examined TLR3/4-induced gene expression. As expected, the enhanced TRIF-dependent *Ifnb* induction observed in LPS-treated *Zfand5*^–/–^ macrophages was completely abolished in *Zfand5*^–/–^*Trif*^–/–^ cells (Supplementary Fig. 6). In contrast, expression of the MyD88-dependent and TRIF-independent gene *Cxcl2* [47] was comparable among WT, *Zfand5*^–/–^, and *Zfand5*^–/–^*Trif*^–/–^ cells. Similarly, the enhanced poly(I:C)-induced *Ifnb* expression observed in *Zfand5*^–/–^ macrophages was also completely abolished in *Zfand5*^–/–^*Trif*^–/–^ cells. Collectively, these results further support a TRIF-dependent mechanism underlying the regulatory role of Zfand5 in TLR3/4-TRIF signaling.

To confirm Zfand5’s role in TRIF regulation, we reconstituted *Zfand5*^–/–^ RAW 264.7 macrophages with Zfand5. Reconstitution of Zfand5 into Zfand5-deficient cells attenuated enhanced TLR4-induced signaling as well as cytokine and IFNβ mRNA expression (Supplementary Fig. 7), further supporting Zfand5’s role in regulating TLR3/4-mediated immune response. Moreover, restoration of Zfand5 also led to a reduction in TRIF protein levels, whereas MyD88 levels remained unchanged (Fig. 3k–m), reinforcing the specificity of Zfand5 for TRIF. Moreover, treatment with the proteasome inhibitor MG132 blocked Zfand5-mediated TRIF degradation in response to LPS, without altering MyD88 levels (Fig. 3n, o). In contrast, inhibition of lysosomal degradation using NH_4_Cl had no effect (Fig. 3p). These results demonstrate that Zfand5 facilitates TRIF degradation via the proteasomal, but not lysosomal, pathway. Taken together, our findings identify Zfand5 as a critical negative regulator of TLR3/4 signaling, functioning by promoting proteasome-dependent degradation of TRIF. This mechanism enables Zfand5 to fine-tune innate immune responses, thereby modulating inflammation and mortality during TLR3/4-driven septic shock.

### Zfand5 associates with TRIF following TLR3/4 activation

**Fig. 4:**
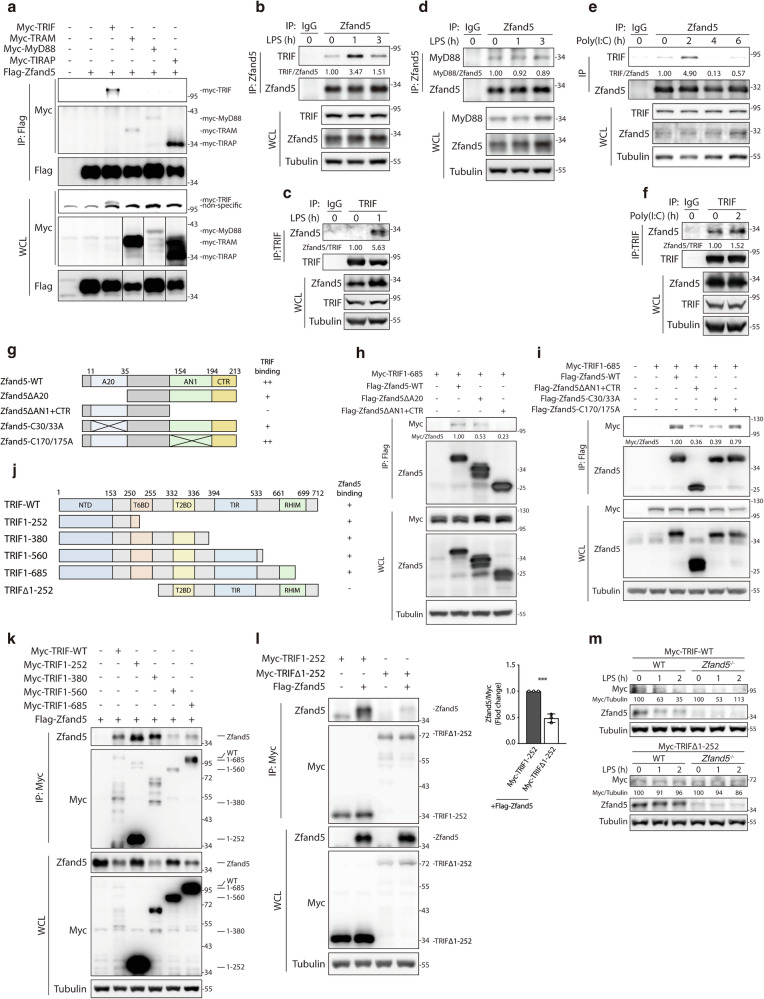
Zfand5 associates with TRIF in response to LPS and poly(I:C) stimulation. **a** HEK293T cells were co-transfected with Flag-tagged Zfand5 and Myc-tagged TRIF, MyD88, TRAM, or TIRAP, as indicated. RAW264.7 cells were stimulated with LPS (100 ng/ml) (**b–d**) or poly(I:C) (100 μg/ml) (**e, f**) for the indicated times. Cell lysates were immunoprecipitated with the indicated antibodies. Immunocomplexes, along with whole cell lysates (WCL), were subjected to immunoblotting with the indicated antibodies. **g** Schematic diagram of full-length Zfand5 and its four mutant forms with an N-terminal Flag tag. **h, i** HEK293T cells were co-transfected with Myc-tagged TRIF(1-685) and either full-length or mutant Flag-tagged Zfand5 constructs. Interactions between TRIF and Zfand5 were identified by immunoprecipitation and immunoblotting with the indicated antibodies. **j** Schematic diagram of full-length TRIF and its five mutant constructs with an N-terminal Myc tag. **k, l** HEK293T cells were co-transfected with Flag-tagged Zfand5 and either full-length or truncated Myc-tagged TRIF variants, as indicated. Interactions between TRIF and Zfand5 were evaluated by immunoprecipitation and immunoblotting with the indicated antibodies. Zfand5 band intensities were normalized to Myc-tagged protein expression and compared to cells expressing Myc-TRIF1-252. Quantification of three independent experiments is shown in the right panel. **m** WT and *Zfand5*^–/–^ RAW264.7 cells were transfected with either wild-type TRIF or TRIFΔ1-252 for 48 h. Cells were pretreated with CHX (2 μg/ml) for 30 mins and then stimulated with LPS (100 ng/ml) for the indicated times. Cell lysates were immunoblotted with the indicated antibodies. Band intensities were normalized to the expression of the control protein and compared to untreated cells, with the results shown below. Statistical significance was determined using Student’s *t* test (***P < 0.01). Data represent three independent experiments and are presented as mean ± SD (error bars).

Previous studies have demonstrated that Zfand5 functions as a proteasome shuttling factor, delivering polyubiquitinated substrates to the proteasome for degradation [36]. Based on these findings and our observations, we hypothesized that Zfand5 specifically interacts with ubiquitinated TRIF protein to promote its proteasome-dependent degradation, thereby modulating TLR3/4 signaling. To test this hypothesis, we first examined whether Zfand5 associates with TRIF using reciprocal co-immunoprecipitation (co-IP). As shown in Fig. 4a, Zfand5 is strongly associated with TRIF, as well as MyD88, but only weakly with other TLR adaptor proteins such as TRAM and TIRAP. Notably, stimulation with LPS or poly(I:C) in RAW 264.7 macrophages significantly enhanced the endogenous interaction between Zfand5 and TRIF (Fig. 4b, c, e, f), whereas Zfand5-MyD88 binding remained unchanged following LPS stimulation (Fig. 4d). To identify the domains responsible for the interaction between Zfand5 and TRIF, we constructed four Zfand5 mutants (Fig. 4g) and five truncated TRIF variants (Fig. 4j). Co-IP experiments demonstrated that deletion of the A20 zinc-finger domain (Δ20) partially reduced the interaction between TRIF and Zfand5, whereas deletion of the AN1 and CTR domains (ΔAN1 + CTR) completely abolished Zfand5’s ability to bind to TRIF (Fig. 4h). To further examine the role of Zfand5’s zinc-finger domains, we generated point mutants that disrupt zinc coordination: C30/33 A mutation in the A20 domain and C170/175 A in the AN1 domain (Fig. 4g). Similar to the A20-deletion mutant, the C30/33 A mutation in the A20 domain partially impaired Zfand5-TRIF binding, while the C170/175 A mutation in AN1 domain had no significant effect (Fig. 4i). These results suggest that both the A20 zinc-finger domain and the CTR region are both essential for Zfand5-TRIF interaction. Mapping of TRIF domains revealed that TRIF(1-252) is sufficient to bind Zfand5, whereas TRIF(253-701) lost this ability (Fig. 4k, l), indicating that Zfand5 associates with the N-terminal region (1-252) of TRIF.

**Fig. 5:**
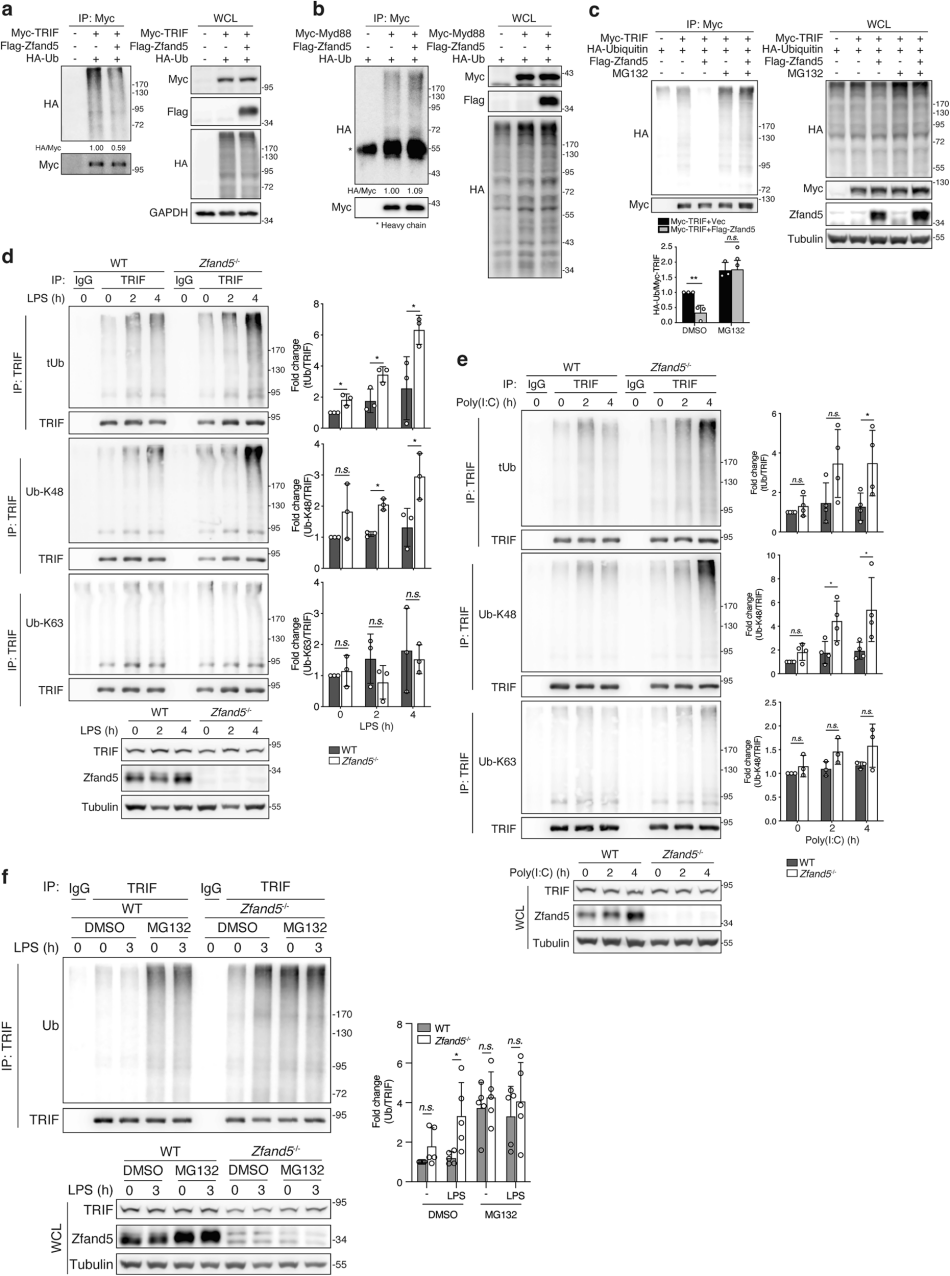
Zfand5 promotes the delivery of ubiquitinated TRIF to the proteasome for degradation following TLR3/4 activation. HEK293T cells were co-transfected with either an empty vector or Flag-tagged Zfand5, along with Myc-tagged TRIF (**a**) or MyD88 (**b**), and HA-tagged Ubiquitin. Ubiquitinated TRIF or MyD88 was assessed by immunoprecipitation of Myc-tagged TRIF or MyD88, followed by immunoblotting with anti-HA antibodies. Band intensities are presented as fold changes relative to vector control cells after normalization to Myc expression, and are shown below. **c** HEK293T cells were co-transfected as in (**a**), and treated with or without MG132 (2 μM) for 6 h. TRIF ubiquitination was determined as in (**a**). **d, e** WT and *Zfand5*^–/–^ RAW264.7 cells were stimulated with LPS (100 ng/ml) or poly(I:C) (100 μg/ml) for the indicated times. **f** WT and *Zfand5*^–/–^ RAW264.7 cells were pretreated with MG132 (2 μM) for 30 mins, followed by stimulation with LPS (100 ng/ml) for the indicated times. TRIF ubiquitination was determined by immunoprecipitating TRIF and subsequent immunoblotting with anti-ubiquitin antibodies. Band intensities are presented as fold increases relative to untreated controls after normalization to TRIF expression. Quantification of three independent experiments is shown in the right panel. Statistical significance was determined using Student’s *t* test (*P < 0.05, **P < 0.01; ns not significant). Data are representative of three independent experiments and are presented as mean ± SD (error bars).

To evaluate the functional importance of Zfand5 domains in TRIF degradation and signaling, we reintroduced wild-type, ΔA20, and ΔAN1 + CTR Zfand5 mutants into *Zfand5*^–/–^ RAW 274.7 cells. Only wild-type Zfand5 restored TRIF degradation in response to LPS and poly(I:C) stimulation, whereas both ΔA20 and ΔAN1 + CTRs deletion mutants failed to do so (Supplementary Fig. 8a, b). Consistently, reintroduction of wild-type Zfand5, but not the mutant forms, reduced the phosphorylation of IKKα/β, TBK1, and IRF3 in response to LPS (Supplementary Fig. 8c). These results indicate that both the A20 domain and the CTR are functionally required for Zfand5-mediated TRIF degradation and downstream signaling.

To confirm whether the binding of Zfand5 to TRIF is necessary for TRIF degradation, we expressed TRIFΔ1-252, which lacks Zfand5 binding ability, in both wild-type and *Zfand5*^–/–^ RAW 264.7 macrophages. As shown in Fig. 4m, wild-type TRIF protein was degraded in wild-type cells but not in *Zfand5*^–/–^ cells following LPS stimulation. In contrast, TRIFΔ1-252 remained stable in both wild-type and *Zfand5*^–/–^ cells upon LPS stimulation, indicating that TRIF degradation is dependent on its ability to bind Zfand5. Taken together, these results demonstrate that Zfand5 directly interacts with TRIF and promotes its proteasome-dependent degradation following TLR3/4 activation.

### Zfand5 facilitates K48-linked polyubiquitination of TRIF for proteasomal degradation following TLR3/4 activation

Ubiquitination is a key post-translational mechanism that regulates TRIF protein stability. Several E3 ubiquitin ligases, including WWP2, TRIM38, and TRAF3, have been shown to promote K48-linked polyubiquitination of TRIF, thereby targeting it for proteasomal degradation [33, 34, 48]. To determine whether Zfand5 regulates the levels of TRIF ubiquitination, we co-transfected HEK293T cells with Flag-tagged Zfand5 and HA-tagged ubiquitin and assessed the levels of ubiquitinated TRIF. As shown in Fig. 5a Zfand5 overexpression significantly reduced TRIF ubiquitination compared to vector-transfected controls. In contrast, Zfand5 had minimal impact on MyD88 ubiquitination, and in some instances even enhanced it (Fig. 5b), suggesting substrate specificity. Importantly, the Zfand5-mediated reduction in TRIF ubiquitination was reversed by treatment with the proteasome inhibitor MG132 (Fig. 5c), indicating that Zfand5 likely functions by delivering ubiquitinated TRIF to the proteasome for degradation. To evaluate the physiological relevance of this mechanism, we examined TRIF ubiquitination in *Zfand5*^–/–^ RAW 264.7 macrophages following LPS and poly(I:C) stimulation. The level of TRIF ubiquitination was significantly elevated in Zfand5-deficient cells compared to wild-type controls (top panels of Fig. 5d, e). Additionally, Zfand5 preferentially influenced K48-linked polyubiquitination of TRIF (middle panels of Fig. 5d, e), while having minimal impact on K63-linked polyubiquitination (bottom panels of Fig. 5d, e) following LPS and poly(I:C) treatment. Consistent with the results in Fig. 5c, treatment of wild-type macrophages with MG132 led to a marked accumulation of ubiquitinated TRIF, reaching levels comparable to those observed in *Zfand5*^–/–^ cells (Fig. 5f), supporting the involvement of Zfand5 and proteasome function within the same proteolytic degradation pathway. Collectively, our findings demonstrate that Zfand5 promotes K-48-linked polyubiquitinated TRIF for proteasomal degradation following TLR3/4 activation.

### Zfand5 promotes the shuttling of TRIF to the proteasome following TLR3/4 activation

**Fig. 6:**
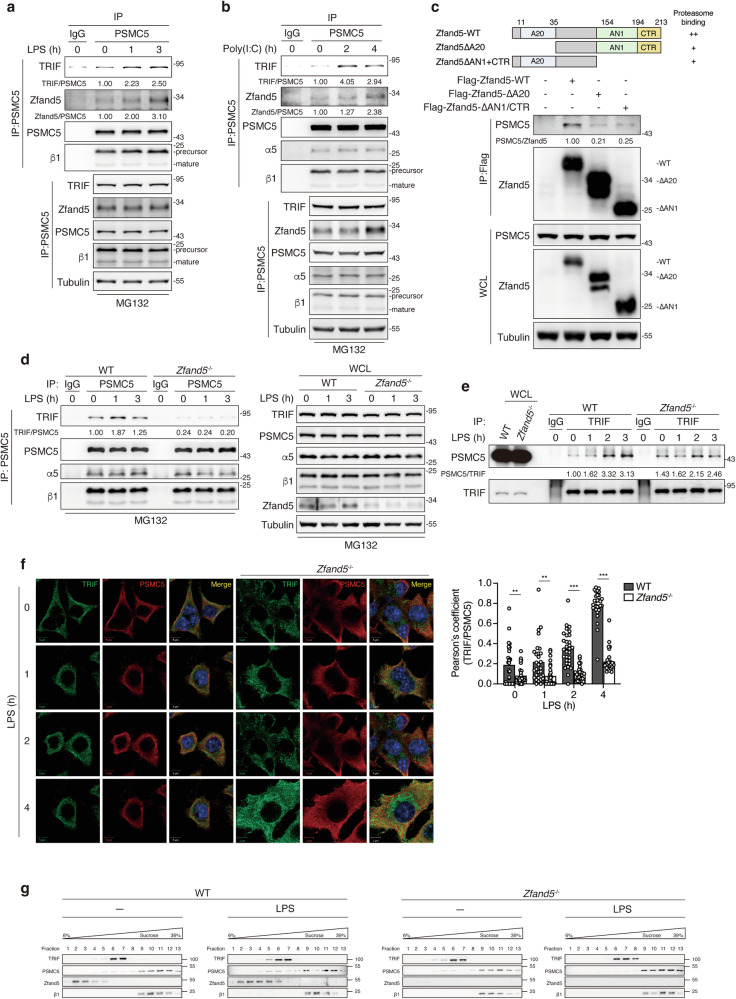
Zfand5 is essential for the interaction between TRIF and the proteasome after TLR3/4 activation. RAW264.7 cells were pretreated with MG132 (2 μM) for 30 mins and then stimulated with LPS (100 ng/ml) (**a**) or poly(I:C) (100 μg/ml) (**b**) for the indicated times. Cell lysates were immunoprecipitated with the indicated antibodies. Immunocomplexes, along with WCL, were subjected to immunoblotting with the indicated antibodies. Band intensities were quantified as fold changes relative to untreated controls after normalization to PSMC5 expression, and are shown below. **c** HEK293T cells were transfected with Flag-tagged full-length Zfand5 or Zfand5 mutants, as indicated. Interactions between PSMC5 and Zfand5 were assessed by immunoprecipitation and immunoblotting with the indicated antibodies. Band intensities were quantified as fold changes relative to Flag-tagged full-length Zfand5-expressing cells after normalization to Zfand5 expression, and are shown below. **d, e** WT and *Zfand5*^–/–^ RAW264.7 cells were pretreated with MG132 (2 μM) for 30 mins and then stimulated with LPS (100 ng/ml) for the indicated times. Cell lysates were immunoprecipitated with the indicated antibodies. Immunocomplexes, along with WCL, were subjected to immunoblotting with the indicated antibodies. Band intensities are presented as fold changes relative to untreated controls after normalization to PSMC5 (**d**) or TRIF (**e**) expression, and are shown below. Data are representative of three independent experiments. **f** *Zfand5*^+/+^ and *Zfand5*^–/–^ BMDMs were treated with LPS (100 ng/ml) for the indicated times. Cells were co-stained with antibodies against TRIF and PSMC5. Quantification of colocalization between TRIF and PSMC5 is shown in the right panel. The colocalization coefficient was quantified using Zen software from at least three independent images (30 cells total). **g** WT or *Zfand5*⁻/⁻ RAW264.7 were pretreated with MG132 (2 μM) for 30 mins and then stimulated with LPS (100 ng/ml) for 3 h. Cell lysates were subjected to sucrose gradient ultracentrifugation to separate proteasome-containing fractions. Gradient fractions were collected sequentially and analyzed by immunoblotting with the indicated antibodies to assess the distribution and composition of proteasome complexes. Statistical significance was determined using Student’s *t* test (**P < 0.01, ***P < 0.001). Data are representative of two independent experiments and presented as mean ± SD (error bars).

Based on the previous reports indicating that Zfand5 functions as a proteasome shuttling factor [36], we investigated whether Zfand5 promotes the recruitment of TRIF to the proteasome following TLR3/4 activation. To test this, we performed co-IP experiments using an anti-PSMC5 antibody, which targets a subunit of the 19S proteasome regulatory particle, to immunoprecipitate the proteasome complex and examine its association with Zfand5 and TRIF. The precipitated proteasome complexes contained the α5 and β1 subunits of the 20S core particle. Following LPS and poly(I:C) stimulation in RAW 264.7 macrophages, we observed a time-dependent increase in the association of both Zfand5 and TRIF with the proteasome (Fig. 6a, b). To determine which domains of Zfand5 mediate its interaction with the proteasome, we co-transfected HEK293T cells with wild-type Zfand5, the ΔA20 mutant, or the ΔAN1 + CTR mutant, and assessed their association with the proteasome. Deletion of the A20 domain or the AN1 plus CTR domains nearly abolished Zfand5’s interaction with the proteasome, indicating that these regions are essential for proteasome binding (Fig. 6c). We next determined whether Zfand5 is required for TRIF-proteasome association by conducting co-IP experiments in wild-type and *Zfand5*^–/–^ RAW 264.7 cells following LPS stimulation. The results revealed that TRIF association with the proteasome was markedly reduced in *Zfand5*^–/–^ cells compared to wild-type controls upon LPS stimulation (Fig. 6d, e). Consistent with these findings, immunofluorescence staining revealed co-localization of TRIF with PSMC5 in wild-type cells after LPS treatment, whereas this co-localization was largely absent in *Zfand5*^–/–^ cells (Fig. 6f). To further examine whether Zfand5 facilitates recruitment of TRIF to the proteasome following LPS stimulation, we performed sucrose gradient ultracentrifugation to isolate and separate proteasome complexes as previously described [49]. In wild-type RAW264.7 cells, LPS stimulation induced co-fractionation of TRIF and Zfand5 with PSMC5 in the same gradient fractions, whereas LPS-induced co-fractionation of TRIF with PSMC5 was markedly reduced in *Zfand5*^–/–^ cells (Fig. 6g). These results suggest that Zfand5 promotes the association of TRIF with the 19S proteasome regulatory particle following LPS stimulation. Taken together, these data identify Zfand5 as a critical mediator facilitating the delivery of TRIF to the proteasome for degradation in response to TLR3/4 activation.

### Zfand5 inhibits TRIF-dependent necroptosis triggered by TLR3/4 activation

**Fig. 7:**
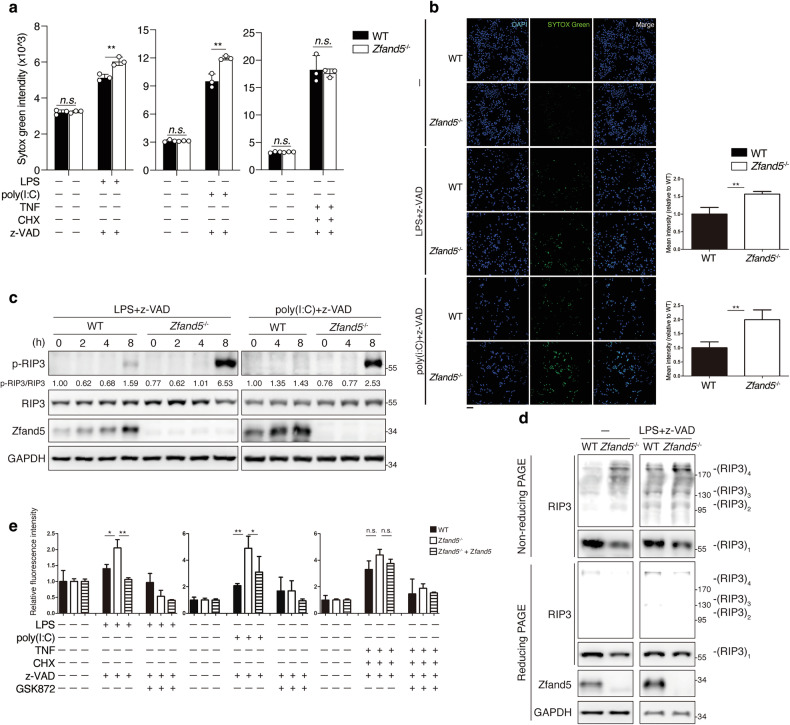
Zfand5 is involved in TLR3/4-, but not TNF-induced, necroptotic cell death. WT and *Zfand5*^–/–^ RAW 264.7 cells were pretreated with z-VAD-fmk (40 μM), alone or in combination of CHX (2 μg/ml) for 30 mins, followed by stimulation with LPS (100 ng/ml), poly(I:C) (30 μg/ml), or TNF (10 ng/ml) for 16 h. Cell death was assessed by staining with Sytox Green (1 μM), and fluorescence intensity was measured using a 96-well plate reader (Victor X4, PerkinElmer) (**a**). Representative Sytox Green images are shown (**b**). The mean fluorescence intensity was quantified using ZEN software from at least three independent images (30 cells total) and is showed in the right panel. **c** WT and *Zfand5*^–/–^ RAW 264.7 cells were pretreated with z-VAD-fmk (40 μM) for 30 mins and stimulated with LPS (100 ng/ml) or poly(I:C) (30 μg/ml) for the indicated times. Cell lysates were analyzed by immunoblotting for phosphorylation of RIPK3 and other indicated proteins. Band intensities were normalized to their unphosphorylated forms and are shown below. **d** WT and *Zfand5*^–/–^ RAW 264.7 cells were pretreated with z-VAD-fmk (40 μM) for 30 mins and then stimulated with poly(I:C) (30 μg/ml) for 8 h. Cell lysates were analyzed by immunoblotting under both non-reducing and reducing SDS-PAGE conditions using the indicated antibodies. **e** WT, *Zfand5*^–/–^ RAW 264.7, and *Zfand5*^–/–^ RAW 264.7 cells reconstituted with Flag-tagged Zfand5 were pretreated with z-VAD-fmk (40 μM), alone or in combination with the RIPK3 inhibitor GSK-872 (10 μM) and CHX (2 μg/ml) for 30 mins, followed by stimulation with LPS (100 ng/ml), poly(I:C) (30 μg/ml), or TNF (10 ng/ml) for 16 hours. Cell death was assessed by staining with Sytox Green (1 μM), and fluorescence intensity was measured using a 96-well plate reader. Statistical significance was determined by Student’s t-test (*P < 0.05, **P < 0.01; ns not significant). Data are representative of two independent experiments performed in triplicate and are presented as mean ± SD (error bars).

Besides driving the production of inflammatory cytokine, chemokine, and type I IFNs, TLR3 and TLR4 can also trigger necroptosis, an inflammatory and lytic form of regulated cell death [50]. TLR3/4-induced necroptosis is mediated through a RIP homotypic interaction motif (RHIM)-dependent interaction between TRIF and receptor-interacting protein kinase 1 (RIPK1) and/or RIPK3, and is typically triggered under conditions of caspase inhibition, such as treatment with the pan-caspase inhibitor z-VAD-fmk. This interaction leads to phosphorylation of mixed lineage kinase domain-like protein (MLKL), which subsequently oligomerizes and inserts into the plasma membrane to execute necroptotic cell death [51]. Given the regulatory role of Zfand5 in TRIF protein stability, we examined its involvement in TLR3/4-triggered necroptotic cell death. In the presence of the pan-caspase inhibitor z-VAD-fmk, both LPS and poly(I:C) induced cell death (Fig. 7a, b), and this effect was augmented in *Zfand5*^–/–^ RAW 264.7 cells. In contrast, TNF-induced necroptosis triggered by TNF in combination with the protein synthesis inhibitor cycloheximide (CHX) and z-VAD-fmk, which proceeds via a TRIF-independent pathway, was minimally affected by Zfand5 deficiency, suggesting that Zfand5 primarily regulates TRIF-dependent necroptotic signaling. To further investigate this, we assessed RIPK3 activation by immunoblotting. Upon LPS or poly(I:C) stimulation in the presence of z-VAD-fmk, phosphorylation (Fig. 7c) and oligomerization of RIPK3, as examined by non-reducing polyacrylamide electrophoresis (PAGE) (Fig. 7d), were markedly enhanced in *Zfand5*^–/–^ RAW 264.7 cells compared to their wild-type counterparts. Furthermore, Zfand5-mediated LPS- and poly(I:C)-induced necroptosis was completely abolished by the RIPK3 inhibitor GSK-872 or by reconstitution of Zfand5 expression (Fig. 7e). These findings indicate that Zfand5 deficiency amplifies RIPK3 activation during TLR3/4-driven necroptotic signaling, likely through dysregulated TRIF protein stability.

Overall, these findings underscore the pivotal role of Zfand5 in regulating TLR3/4-driven innate immune responses and reveal a previously unrecognized mechanism controlling TRIF-dependent necroptotic signaling.

## Discussion

**Fig. 8:**
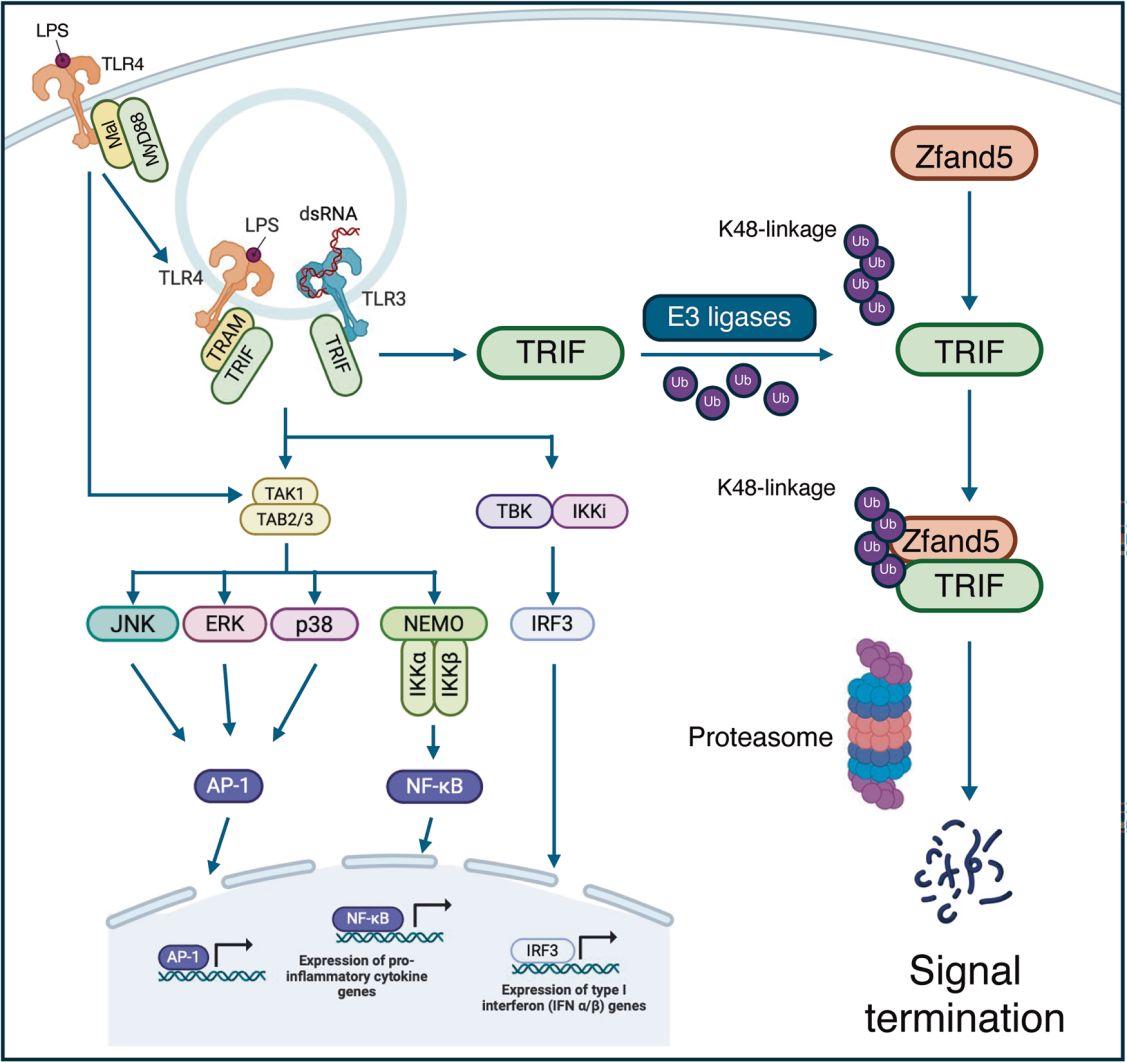
Graphical summary of Zfand5-mediated regulation of TRIF degradation through the proteasomal pathway. Upon stimulation with LPS or poly(I:C), TLR4 initiates MyD88-dependent signaling at the plasma membrane and is subsequently internalized into endosomes, where it engages TRIF, whereas endosomal TLR3 directly recruits TRIF. TRIF-dependent signaling downstream of both TLR3 and TLR4 activates IRF3, NF-κB, and MAPKs. Zfand5 binds to K48-linked polyubiquitinated TRIF and facilitates its delivery to the proteasome for degradation, thereby attenuating both TLR3/TRIF- and TLR4/TRIF-mediated signaling. In the absence of Zfand5, TRIF degradation is impaired, resulting in TRIF accumulation, prolonged signaling, and increased production of inflammatory cytokines and type I interferons (IFNs).

TLR-mediated inflammatory responses play a fundamental role in initiating host defense mechanisms, including pathogen elimination and the activation and shaping of adaptive immunity [52]. However, these responses require strict regulation to prevent excessive inflammation and tissue damage [18]. Disruption of this balance can lead to chronic inflammation or autoimmune disorders, underscoring the importance of precise control of TLR signaling in maintaining immune homeostasis [17, 18]. Despite extensive research, the molecular mechanisms regulating TLR-driven immune responses remain incompletely understood. In this study, we identified Zfand5, a zinc finger-containing protein, as a previously unrecognized regulator of TLR3/4-mediated immune responses. Our data demonstrate that Zfand5 promotes proteasomal degradation of the adaptor protein TRIF, thereby selectively suppressing TLR3/4-induced inflammation (Fig. 8). We further show that Zfand5 facilitates delivery of ubiquitinated TRIF to the proteasome for degradation, leading to termination of TLR3/4 signaling. Functionally, this regulation reduces the production of pro-inflammatory cytokines and type I interferons in response to TLR3/4 agonists and uropathogenic *E. coli*, and protects against TLR3/4-driven sepsis. Together, our findings uncover a distinct regulatory mechanism that selectively limits TRIF-dependent inflammatory signaling.

Zfand5 is a member of the Zfand protein family, many of which contribute to proteasome-mediated degradation and protein homeostasis under various physiological conditions [53]. For instance, Zfand1 recruits the ubiquitin-selective ATPase p97 and the 26S proteasome to clear arsenite-induced stress granules, while Zfand5 has been shown to transiently interact with the proteasome and ubiquitinated substrates to facilitate their degradation [38, 54]. Our results reveal that Zfand5 binds directly to both ubiquitinated TRIF and the proteasome, thereby enabling TRIF degradation. These findings provide evidence that a Zfand family protein can mediate the selective turnover of a signaling adaptor. Previous structural studies have shown that Zfand5’s A20 domain binds ubiquitinated proteins, while its AN1 domain enhances proteasomal peptidase activity [37, 38]. Cryo-EM analyses further suggest that Zfand5 most efficiently delivers ubiquitinated substrates to the proteasome via its A20 domain and C-terminal region (CTR), with the A20 zinc finger domain recognizing polyubiquitin chains and the CTR stabilizing Zfand5-substrate interactions. Consistent with this, our domain-mapping analysis revealed that both the A20 and CTR domains are essential for Zfand5-TRIF binding, supporting a model in which Zfand5 identifies ubiquitinated TRIF and guides it to the proteasome for degradation.

Zfand5 was originally identified as an IKKγ-interacting protein and has been shown to associate with several immune regulators, including TRAF6 and RIP [41]. Overexpression studies have suggested that Zfand5 can inhibit NF-κB activity [41], and additional research has shown that it acts as a suppressor of osteoclast differentiation independent of NF-kB signaling [55]. Its close homolog, Zfand6, has been implicated in TRAF2-mediated NF-κB signaling, though its overexpression similarly suppresses NF-κB activation [56, 57]. Despite these findings, the physiological relevance of Zfand5 and Zfand6 in innate immunity has remained unclear. Our results demonstrate that Zfand5 regulates TLR3- and TLR4-mediated NF-κB activation primarily by controlling the stability of their adaptor protein TRIF. In contrast, Zfand5 has minimal impact on TLR2-, TLR7-, or TLR9-driven NF-κB activation, and Zfand6 does not influence TLR4 signaling. These findings suggest that neither protein directly modulates NF-κB transcriptional activity, but instead influences upstream adaptor stability in a pathway-specific manner.

Various regulatory mechanisms have been described in TLR signaling, including compartmentalization and ligand availability, phosphorylation/dephosphorylation, post-translational modification (such as ubiquitination and cleavage), and negative feedback regulation [10, 58–60]. Numerous negative regulators have been identified that function at different levels to maintain immune balance. For example, deubiquitinating enzymes A20 and cylindromatosis (CYLD) remove ubiquitin chains from TRAF6 to inhibit MyD88-dependent signaling [61, 62], while deubiquitinating enzyme A (DUBA) targets TRAF3 to suppress TRIF-dependent type I IFN production [63]. TIR domain-containing adaptors are also common regulatory targets to enable rapid signal termination [10]. For instance, tyrosine kinase Syk and C-type lectin receptor-2d (CLEC2D) promote MyD88 degradation [23, 64], and SOCS1 reduces TIRAP protein stability, suppressing TLR2- and TLR4-induced NF-κB activation [65]. Our study adds Zfand5 to this group of negative regulators, identifying it as a key factor that specifically targets TRIF for degradation and helps terminate TLR3/4 signaling.

TRIF is a central adaptor downstream of TLR3 and TLR4 that mediates endosomal signaling to induce type I interferon production, NF-κB activation, and necroptosis [51, 66, 67]. Although TRIF-dependent signaling is initiated at endosomal membranes, this does not necessarily imply that TRIF turnover is governed exclusively through endo-lysosomal degradation pathways. Endosomes primarily function as signaling platforms that spatially organize TLR3/4 and their adaptor TRIF, whereas endo-lysosomal degradation typically targets membrane-associated receptors or cargo marked by K63-linked polyubiquitin chains following signal termination [19]. In contrast, TRIF has been reported to undergo K48-linked polyubiquitination and subsequent proteasomal degradation following TLR3/4 activation [23, 34, 35], indicating that endosome-activated TRIF can also be eliminated through the ubiquitin-proteasome system. Our data demonstrate that Zfand5 binds polyubiquitinated TRIF and selectively delivers it to the proteasome rather than routing it to lysosomes, highlighting a critical role for Zfand5 in facilitating proteasomal degradation of TRIF downstream of endosomal signaling. Thus, while endosomal localization is essential for TRIF activation, proteasome-mediated degradation represents a mechanistically distinct downstream checkpoint that ensures timely termination of TLR3/4-driven inflammatory responses.

While TRIF-mediated signaling can be protective during microbial infections, excessive activation—especially through TLR3 or TLR4—has been linked to pathological inflammation and diseases such as steatosis, fibrosis, and chronic liver injury [68, 69]. Thus, precise regulation of TRIF signaling is critical to preventing immune overactivation and maintaining homeostasis. Although MyD88 regulation has been extensively studied, mechanisms governing TRIF stability and degradation remain poorly understood. Our study shows that Zfand5 mediates TRIF degradation through the proteasome in response to LPS and poly(I:C). Consistent with the fact that mammalian Zfand proteins, except Zfand7, lack enzymatic activity [53], Zfand5 likely functions as a shuttling factor that couples ubiquitinated TRIF to the degradation machinery. Zfand5 deficiency leads to the accumulation of ubiquitinated TRIF and reduced TRIF-proteasome interaction, further supporting an essential role for Zfand5 in facilitating TRIF turnover.

K48-linked polyubiquitination is a key signal for targeting TRIF for proteasomal degradation. Several E3 ubiquitin ligases, including Cbl-b [23], TRAF3 [33], WWP2 [34], and TRIM38 [35], have been implicated in this process. We found that Zfand5 binds to the N-terminal region of TRIF (residues 1-252), which includes lysine 228 (K228), a site previously shown to be ubiquitinated by TRIM38 [35, 48]. Consistently, a TRIF mutant lacking this region fails to undergo degradation upon TLR4 activation, highlighting its importance in TRIF ubiquitination and turnover. These findings suggest that Zfand5 may serve as a key regulator of TRIF homeostasis during TLR3/4 activation, as knockdown of Zfand5 leads to increased stimulus-induced TRIF ubiquitination while reducing its proteasomal degradation. Although Zfand5 promotes TRIF degradation, it reduces the steady-state level of detectable ubiquitinated TRIF. This is consistent with a model in which Zfand5 acts downstream of TRIF ubiquitination by associating with ubiquitinated TRIF and facilitating its delivery to the proteasome, thereby promoting rapid turnover of this pool. Accordingly, ubiquitinated TRIF accumulates in Zfand5-deficient cells due to impaired proteasomal clearance, whereas it is efficiently degraded in wild-type cells. Notably, in the presence of the proteasome inhibitor MG132, ubiquitinated TRIF levels become comparable between wild-type and Zfand5-deficient macrophages following TLR4 activation, further supporting a role for Zfand5 in proteasomal targeting rather than ubiquitin conjugation. However, we cannot exclude the possibility that Zfand5 may also influence the efficiency or dynamics of TRIF ubiquitination.

During infection, PRRs detect PAMPs and rapidly initiate innate immune responses to combat invading microbes [1–3]. Once the pathogen threat is resolved, inflammatory signaling must be quickly downregulated to prevent host damage. Various mechanisms have evolved to terminate PRR-driven responses [9, 10, 53]. Our findings establish that Zfand5 negatively regulates TLR3/4 signaling by promoting TRIF degradation. Given TRIF’s essential role in activating NF-κB, mediating IRF3-driven type I interferon production, and triggering RIPK3-dependent necroptosis [51, 66, 67], its targeted degradation facilitates the timely resolution of inflammatory signaling. Combined with the inherently short half-lives of many cytokine mRNAs, this mechanism contributes to the rapid shutdown of immune activation. Zfand5 has also been reported to enhance proteasomal activity [37]. Consistent with this, our results demonstrate that TLR3/4 stimulation promotes the interaction between Zfand5 and TRIF, but not with MyD88. As levels of ubiquitinated proteins increase during inflammation, Zfand5 may function both as a selective shuttling factor and as a proteasome activator to ensure efficient degradation of TRIF. Notably, although Zfand5 expression is induced following LPS stimulation, inflammatory mediators continue to increase during the early phase of stimulation. This apparent discrepancy likely reflects the temporal organization of TLR signaling, in which rapid pathway activation is required for effective host defense, whereas negative regulators function primarily to restrain the magnitude and duration of responses rather than prevent their initiation. Moreover, LPS activates both MyD88- and TRIF-dependent pathways, and thus suppression of TRIF stability would be expected to preferentially dampen the late-phase TRIF-driven type I IFN production while allowing early MyD88-dependent inflammatory gene induction to proceed. Therefore, Zfand5 likely functions as a delayed negative-feedback regulator that promotes resolution of TLR3/4-driven inflammation after an initial protective response has been established.

TRIF-dependent signaling has been implicated in sepsis, tissue injury, and chronic inflammatory diseases [51, 66, 67], suggesting that modulation of the Zfand5-TRIF axis may have therapeutic potential. Enhancing Zfand5 activity or promoting TRIF proteasomal turnover could represent a strategy to attenuate excessive TLR3/4-driven inflammation during endotoxemia or bacterial sepsis. Conversely, transient inhibition of Zfand5-mediated TRIF degradation may be beneficial in settings where amplified type I IFN responses are desirable, such as antiviral immunity or vaccine adjuvant responses. Future studies examining Zfand5 expression and genetic variation in human inflammatory diseases, as well as evaluating pharmacological modulation of Zfand5-proteasome interactions, will be important for determining the clinical relevance of this regulatory pathway.

Several limitations of this study should be noted. First, although our data demonstrate that Zfand5 promotes proteasome-dependent degradation of ubiquitinated TRIF, the upstream E3 ubiquitin ligase(s) responsible for TRIF ubiquitination in our experimental system remain to be fully defined. We also cannot exclude the possibility that Zfand5 may facilitate TRIF ubiquitination in addition to shuttling ubiquitinated TRIF to the proteasome for degradation. Second, the molecular mechanisms governing Zfand5 induction following TLR activation, as well as the structural determinants underlying its specificity for ubiquitinated TRIF, require further investigation. Third, although sucrose gradient ultracentrifugation analysis revealed that Zfand5 promotes association of TRIF with the 19S proteasome regulatory particle, the transient nature of proteasome-substrate interactions prevents us from excluding the possibility that Zfand5 facilitates direct binding of TRIF to the intact 26S proteasome complex. Addressing these questions will provide deeper mechanistic insight into TRIF homeostasis and may facilitate the development of therapeutic strategies targeting Zfand5 to modulate TLR3/4-driven inflammation. More broadly, these findings provide a framework for investigating whether ZFAND5-mediated TRIF turnover contributes to human inflammatory diseases and may guide future efforts to therapeutically modulate TRIF-dependent signaling.

In summary, our study identifies Zfand5 as a critical regulator of TRIF stability and reveals the Zfand5-TRIF axis as a novel and non-redundant mechanism for terminating TLR3- and TLR4-mediated immune responses. Given the essential role of TRIF-mediated signaling in type I interferon production, therapeutic targeting of Zfand5 may represent a promising strategy for enhancing antiviral immunity and improving vaccine efficacy.

## Materials and methods

### Plasmids

An N-terminal Flag-tagged Zfand5 was cloned into the pLKO.AS2 vector (National RNAi Core Facility, Academia Sinica, Taiwan). Plasmids encoding Zfand5-C30/33 A and Zfand5-C170/175 A mutants were generated via site-directed mutagenesis using the QuickChange II Site-Directed Mutagenesis Kit (Stratagene, La Jolla, CA, USA). The constructs pEF4-Flag-Zfand5ΔΑ20 (amino acids 36-213) and pEF4-Flag-Zfand5Δ(AN1 + CTR) (amino acids 1-153) were generously provided by Dr. Ken Watanabe (National Center for Geriatrics and Gerontology, Japan) [55]. Flag-tagged Zfand5, Zfand5Δ20, and Zfand5Δ(AN1 + CTR) were subsequently subcloned into the pAS4.1w.Pbsd-aOn vector (National RNAi Core Facility, Academia Sinica, Taiwan). The pRK5-myc-TRIF construct was previously described [70]. TRIF mutant derivatives were generated by PCR amplification using pRK5-myc-TRIF as the template and subsequently subcloned into the pRK5 vector. The pcDNA-HA-Ubiquitin plasmid was obtained from Addgene (Cambridge, MA, USA). The pMD.G, and pCMVΔR8.91 plasmids were sourced from the National RNAi Core Facility, Academia Sinica, Taiwan.

### Reagents and antibodies

A custom anti-Zfand5 polyclonal antibody (1:10,000 dilution for immunoblotting, 1:500 for immunoprecipitation) was generated by immunizing rabbits with a recombinant mouse Zfand5 peptide (amino acid residues 53-152), which was expressed in *Escherichia coli* BL21 and subsequently affinity-purified using Ni-NTA agarose beads (Qiagen, Hilden, Germany), according to the manufacturer’s instructions. Rabbit antisera were collected and further purified using Protein A agarose beads (Merck Millipore, Billerica, MA, USA) following the manufacturer’s instructions. Antibodies against phospho-IKKα/β (#2697, 1:1000), phospho-TBK1 (#5483, 1:1000), TBK1 (#3504, 1:1000), phospho-IRF3 (#4947, 1:1000), IRF3 (#4302, 1:1000), phospho-p38 (#9211, 1:1000), p38 (#2912, 1:1000), phospho-JNK1/2 (#9251, 1:1000), JNK1/2 (#9252, 1:1000), phospho-ERK1/2 (#4370, 1:1000), ERK1/2 (#9102, 1:1000), phospho-RIP3 (#57220, 1:1000), RIP3 (#95702, 1:1000), MLKL (#26539, 1:1000) were purchased from Cell Signaling Technology (Danvers, MA, USA). Anti-IKKα/β (#sc-7607, 1:1000), IκBα (#sc-371, 1:1000), MyD88 (#sc-11356, 1:1000), and Ubiquitin (#sc-8017, 1:1000) antibodies were obtained from Santa Cruz Biotechnology (Santa Cruz, CA, USA). Anti-HA (#ab71113, 1:5000) and phospho-MLKL (#ab196436, 1:1000) antibodies were purchased from Abcam (Cambridge, UK). GAPDH (#GTX627408, 1:10000) antibody was obtained from GeneTex (Irvine, CA). Anti-Ubiquitin-K48 (#05-1307, 1:1000) and Ubiquitin-K63 (#05-1308, 1:1000) antibodies were purchased from Merck Millipore (Billerica, MA, USA). Anti-TRIF (#657102, 1:1000) antibody was purchased from BioLegend (San Diego, CA, USA). Anti-Flag (#F3165, 1:5000) and anti-Myc (#M4439, 1:5000) antibodies, anti-Flag M2 Affinity Gel (#A2220), LPS (#L6529, *E.coli* 055:B5), LPS (#L3024, *E.coli* O111:B4), MG132 (#C2211), Cycloheximide (#C1988) were purchased from Sigma-Aldrich (St. Louis, MO, USA). Tubulin (#C06002-100UL, 1:10000) antibody was purchased from Croyez (Taipei City, Taiwan (R.O.C.)). The secondary antibodies, Peroxidase-conjugated AffiniPure Goat Anti-Rabbit IgG (#111-035-003, 1:5000), Peroxidase-conjugated AffiniPure Goat Anti-Mouse IgG (#115-035-003, 1:5000) were purchased from Jackson Immunoresearch (West Grove, PA, USA). Macrophage colony-stimulating factor (M-CSF) (#315-02) and fms-related tyrosine kinase 3 ligand (Flt3L) (#300-19) were purchased from Peprotech (Rocky Hill, NJ, USA). Poly(I:C) (#27-4732-01) and Pam_3_CSK_4_ (#TLRL-PMS) were purchased from GE Healthcare (Little Chalfont, UK) and Invivogen (San Diego, CA, USA), respectively. Puromycin (#P-600) and SYBR Green (#4368706) were purchased from Gold Biotechnology (St. Louis, MO, USA) and ABI (Waltham, MA, USA), respectively.

### Mice

*Zfand5*^F/F^ mice were generated by inserting two loxP sites into the third and fifth introns of the *Zfand5* gene via two homologous recombination events in embryonic stem (ES) cells. To achieve systemic deletion of *Zfand5*, *Zfand5*^F/F^ mice were crossed with *Sox2*-*Cre* transgenic mice, which express Cre recombinase under the control of the SPY-box containing gene 2 (SOX2) promoter [43]. The Sox2 promoter drives Cre recombinase activity in epiblast cells starting at embryonic day 6.5 (E6.5). *Zfand5*^F/F^ mice were bred with *Sox2*-Cre mice to obtain *Zfand5*^F/-^ mice carrying *Sox2*-*Cre*. These offspring were then crossed with C57BL/6 J mice to generate *Zfand5*^+/^ mice, which were further intercrossed to produce *Zfand5*^–/–^ mice. All animal experiments were performed in accordance with animal welfare guidelines and were approved by the Institutional Animal Care and Use Committee (IACUC) of the College of Medicine, National Taiwan University (approval no. 20230002).

### Cell cultures

RAW 264.7 macrophages were cultured in RPMI-1640 (Hyclone, Logan, UT, USA) supplemented with 10% (vol/vol) heat-inactivated fetal bovine serum (FBS) (Corning, New York, USA), 100 U/mL penicillin/streptomycin (Corning) and 1 mM sodium pyruvate (Corning). HEK293T cells and immortalized mouse embryonic fibroblasts (iMEFs), the latter kindly provided by Dr. Chien-Kuo Lee (National Taiwan University, Taiwan) [71], were cultured in DMEM medium (Hyclone) containing 10% (vol/vol) heat-inactivated FBS, 100 U/mL penicillin/streptomycin, and 1 mM sodium pyruvate. All cells were maintained at 37 °C in a humidified atmosphere containing 5% CO_2_.

### Preparation of bone marrow-derived macrophages and dendritic cells

Bone marrow-derived macrophages (BMDMs) were prepared as previously described [72]. Briefly, bone marrow cells were isolated from the femurs and tibiae of 6- to 8-week-old mice by flushing with DMEM medium using a 25-gauge syringe. The collected cells were cultured in high-glucose DMEM medium supplemented with 20% L929 cell-conditioned medium for 7 days to allow differentiation into macrophages. Fully differentiated BMDMs were then collected and maintained in DMEM containing 10 ng/ml M-CSF for subsequent experiments. Bone marrow-derived dendritic cells (BMDCs) were prepared by differentiating bone marrow cells in high-glucose DMEM medium containing 300 ng/ml Flt3L for 7 days, followed by collection for subsequent assays.

### sgRNA- and shRNA-mediated gene silencing and lentiviral infection

Lentiviral sgRNA constructs encoding small guide RNAs (sgRNAs) targeting mouse *Zfand5* (in the pALL-Cas9. Ppuro vector) and lentiviral short hairpin RNA (shRNA) constructs targeting mouse *Zfand6* (in the pLKO-pure vector) were obtained from the National RNAi Core Facility Platform at the Institute of Molecular Biology/Genomic Research Center, Academia Sinica, Taiwan. The target sequences were as follows:

sgRNA target sequences for Zfand5:

Sense guide oligo: 5’-cacc-GTACTACACAGCATGGGCCCT

Antisense guide oligo: 5’-aaac-AGGGCCCATGCTGTGTAGTAC.

shRNA sequence for Zfand6:

5’-TTTCTTTGCACCCACTATTTA-3’.

HEK293T cells were transfected with the lentiviral sgRNA constructs along with packaging plasmids pMD.G and pCMVΔR8.91 using Turbofect transfection reagent (Fermentas, Schwerte, Germany) according to the manufacturer’s protocol. Culture medium containing the lentivirus was collected at 48 and 72 h post-transfection. RAW 264.7 cells and iMEFs were infected overnight with lentiviral supernatants in the presence of 8 μg/ml polybrene (Sigma-Aldrich). After infection, cells were cultured in fresh medium for another 24 h. The infected cells were selected in medium containing 5 μg/ml puromycin until all uninfected cells were completely killed. The resulting puromycin-resistant colonies were pooled and expanded for further experiments.

### RNA purification and quantitative RT-PCR

Total cellular RNA was extracted using NucleoZOL (MACHEREY-NAGEL Inc, Düren, Germany) following the manufacturer’s instructions. One μg of RNA was reverse-transcribed into complementary DNA (cDNA) using the RevertAid H Minus First Strand cDNA Synthesis Kit (Thermo Fisher Scientific, Rockford, IL, USA) according to the manufacturer’s instructions. The amounts of cDNA were quantified using Real-time quantitative PCR (qPCR) with the Maxima SYBR Green/Fluorescein qPCR Master Mix (Thermo Fisher Scientific) according to the manufacturer’s recommendations. All qPCR values were normalized to cyclophilin mRNA levels to determine relative expression. All experiments were conducted in triplicate, and the primer sequences used are listed in Supplementary Table 2.

### Dual-luciferase reporter assay

HEK293T cells were co-transfected with a Firefly luciferase reporter plasmid (pIFNβ-Luc or pNF-κB-Luc), a Renilla luciferase control plasmid (pRL-TK), and other indicated plasmids. After 48 h, cells were lysed, and both Firefly and Renilla luciferase activities were determined using the Dual-luciferase reporter assay system (Promega), according to the manufacturer’s instructions. Firefly luciferase activity was normalized to Renilla luciferase to control for transfection efficiency.

### Immunoblotting and immunoprecipitation

Cells were harvested and lysed in ice-cold lysis buffer containing 150 mM NaCl, 50 mM Tris-HCl pH7.5, 2 mM EDTA, 1% Triton X-100, 0.5% NP-40, 10% Glycerol, protease inhibitors (1 μg/ml of Aprotinin, Benzamidine, Pepstatin A, and Leupeptins from Sigma-Aldrich), 1 mM phenylmethylsulfonyl fluoride (PMSF), 2 mM dithiothreitol, 1 mM sodium orthovanadate (NA_3_VO_4_), and incubated on ice for 30 min. For the detection of ubiquitinated protein, 20 mM N-Ethylmaleimide (#E3876, Sigma-Aldrich) was added to the lysis buffer. Protein concentrations were determined using the Bio-Rad Protein Assay (#5000006, Bio-Rad, Hercules, CA, USA). Proteins were resolved by sodium dodecyl sulfate polyacrylamide gel electrophoresis (SDS-PAGE) and transferred onto a Polyvinylidene fluoride (PVDF) membrane (Merck Millipore, Burlington, MA, USA). The PVDF membrane was blocked with 10% skim milk in TBST (50 mM Tris-HCl, 150 mM NaCl, 0.05% Tween-20, pH 7.6) for 1 h, then incubated overnight at 4 °C with the indicated primary antibody. After washing, the membrane was incubated with HRP-conjugated secondary antibody for 1 h at room temperature. Immunoreactive signals were detected using Western Lightning Plus-ECL (#NEL103001EA, PerkinElmer, Waltham, MA, USA) following the manufacturer’s instructions. For immunoprecipitation, cell extracts were incubated with the appropriate primary antibody overnight at 4 °C, followed by a 2-h incubation with Protein A or Protein G Sepharose beads (Merck Millipore). The immunocomplexes were washed, subjected to SDS-PAGE, and analyzed by immunoblotting.

### Immunofluorescence

Cells were seeded onto coverslips in complete culture medium and allowed to adhere overnight. After stimulation with the indicated ligands, cells were fixed with 4% paraformaldehyde (PFA)(Alfa Aesar, Ward Hill, MA, USA) in phosphate-buffered saline (PBS, pH 7.4) for 15 min at room temperature. Cells were then permeabilized with 0.25% Triton X-100 in PBS for an additional 15 min at room temperature. To block nonspecific binding, cells were incubated with 1% bovine serum albumin (BSA)(Sigma-Aldrich) in PBST (PBS with 0.05% Tween-20) for 30 min at room temperature. Coverslips were then incubated with primary antibodies overnight at 4 °C. Afterward, the coverslips were washed three times with PBS and then incubated with fluorescence-conjugated secondary antibodies for 1 h at room temperature. Following another three washes with PBS, coverslips were mounted using DAPI Fluoromount-G (SouthernBiotech, Birmingham, AL, USA) to counterstain nuclei. Images were captured using an LSM 700 confocal microscope (Zeiss, Oberkochen, Germany) and analyzed with ZEN software (Zeiss) and ImageJ software.

### Enzyme-linked immunosorbent assays (ELISA)

The levels of cytokines in culture supernatants and mouse sera were measured using DuoSet ELISA kits (R&D Systems, Minneapolis, MN, USA) following the manufacturer’s instructions. Briefly, a 96-well plate was coated with the capture antibody and incubated overnight at 37 °C. After coating, the plate was washed three times with PBST (137 mM NaCl, 2.7 mM KCl, 8.1 mM Na_2_HPO_4_, 1.5 mM KH_2_PO_4_, pH 7.2-7.4, and 0.05% Tween-20) and blocked with Reagent Diluent (1% BSA in PBS) for 1 h at room temperature. After three washes with PBST, cultural supernatants, diluted sera, or standardized recombinant proteins were added to the plate and incubated overnight at 4 °C. Following another three washes with PBST, the detection antibody was added to the plate and incubated for 2 h at room temperature, followed by three washes with PBST. Streptavidin-HRP was added to the plate and incubated for 30 min at room temperature, followed by three washes with PBST. The substrate solution (1:1 mixture of H_2_O_2_ and tetramethylbenzidine (TMB)) was added to the plate and incubated for 15–20 min at room temperature. The reaction was stopped with the stop solution (2 N H_2_SO_4_). The optical density of the solution was measured at 450 nm using a VICTOR X4 Multilabel Plate Reader (PerkinElmer).

### Fluorescence necroptotic cell death assay

RAW 264.7 cells were seeded in a 96-well plate at a density of 5000 cells per well one day prior to the assay. Necroptosis was induced by pre-treating cells with either 40 μM z-VAD-fmk (APExBio, Houston, TX, USA) or with 2 μg/ml cycloheximide (CHX) (Sigma-Aldrich) and 40 μM z-VAD-fmk for 30 min, followed by stimulation with 100 ng/ml LPS, 30 μg/ml poly(I:C), or 10 ng/ml TNF (Cell Signaling Technology) for 16 h. Fluorescent signals corresponding to cell death were detected using a 96-well plate reader (Victor X4, PerkinElmer). Data were analyzed and visualized as bar charts generated by GraphPad Prism software.

### Proteosome isolation

Proteasomes were isolated by sucrose gradient ultracentrifugation as previously described [49], with minor modifications. Briefly, cell lysates were layered onto a discontinuous sucrose gradient prepared in PBS, consisting of sucrose solutions ranging from 39% (bottom) to 6% (top) in 3% increments. Lysates were loaded onto the top of the gradient (6% sucrose) and subjected to ultracentrifugation using an SW41 Ti rotor (Beckman, Germany) at 39,000 rpm for 18 h at 4 °C. Following centrifugation, gradient fractions were collected sequentially from the top and analyzed by immunoblotting using the indicated antibodies.

### Mouse model of sepsis

To induce septic shock, age- and weight-matched male *Zfand5*^+/+^ and *Zfand5*^–/–^ mice were intraperitoneally administered either bacterial endotoxin LPS (15 mg/kg) or poly(I: C) (2 mg/kg) in combination with D-galactosamine (D-GalN, 0.5 mg/g, Merck Millipore). Survival rates were monitored for 7 days post-injection. Blood samples were collected 4 h after challenge for cytokine analysis, and lung tissues were harvested for histological examination.

### Statistical analysis

All data are presented as mean ± SD. Significant differences between the two groups were determined by a two-tailed Student’s *t* test. A p-value of less than 0.05 was considered statistically significant.

## Supplementary information

Supplementary Materials

original data

## Data Availability

The data supporting the findings of this study are available from the corresponding author upon reasonable request.
