# Supplementary material for: Zfand5 terminates TLR3/4 signaling and necroptosis by targeting TRIF to the proteasome for degradation
Source: Cell Death Dis. 2026 Jun 5;17(1):704. doi: 10.1038/s41419-026-08946-0 (PMC13458262; doi:10.1038/s41419-026-08946-0)
Supplement: Supplementary file 1 [file 41419_2026_8946_MOESM1_ESM.docx]

**Supplementary Fig. 1. Zfand5 selectively regulates TLR3/4-, but not TLR2/7/9-mediated innate immune responses in RAW 264.7 cells.**
(a, b) Wild-type (WT) and *Zfand5*^-/-^ RAW 264.7 macrophages were treated with LPS (100 ng/ml), poly(I:C) (30 μg/ml), Pam_3_CSK_4_ (100 ng/ml), Zymosan (25 μg/ml), R837 (5 μg/ml), or CpG-A (1 μM) for 4 hours. The mRNA expression of the indicated genes was analyzed by RT-qPCR. (c-e) WT and *Zfand5*^-/-^ RAW 264.7 cells were stimulated with LPS (100 ng/ml), poly(I:C) (30 μg/ml), or Pam_3_CSK_4_ (100 ng/ml) for the indicated times. Phosphorylation of IKKα/β, TBK1, IRF3, and MAPKs, as well as total protein levels, was assessed by immunoblotting. Band intensities are presented as fold changes related to untreated controls after normalization to their unphosphorylated forms, and are shown below. (f-j) WT and *Zfand5*^-/-^ RAW 264.7 cells were treated with LPS (100 ng/ml), poly(I:C) (30 μg/ml), or Pam_3_CSK_4_ (100 ng/ml) for the indicated times. Cells were co-stained with antibodies against NF-κB p65 or IRF3. Nuclear translocation was quantified by colocalization between NF-κB p65 or IRF3 and DAPI, and is shown in the right panels. Colocalization coefficients were determined using Zen software from at least three independent images (≥30 cells). Statistical significance was determined using Student’s *t*-test (***P* < 0.01, ****P* < 0.001; *n.s.*, not significant). Data are representative of two independent experiments and are presented as mean ± SD (error bars).

**Supplementary Fig. 2. Generation and characterization of *Zfand5*-deficient mice.**
(a) Schematic diagram illustrating the mouse wild-type (*Zfand5*) allele, the two targeting constructs, the targeted allele, the floxed (*Zfand5*^Flox^) allele, and the Zfand5-deleted allele following Cre-mediated recombination. (b) Genotypic ratio of offspring resulting from intercrosses of *Zfand5*^+/-^ mice. (c) Body weights of 8-week-old male mice of the indicated genotypes. (d) BMDMs differentiated from bone marrow cells isolated from *Zfand5*^+/+^ and *Zfand5*^-/-^ mice were stained with CD45, CD11b, and F4/80 antibodies, followed by flow cytometric analysis. The percentage of CD45^+^CD11b^+^F4/80^+^ BMDMs is shown in the right panel. (e) Average number of BMDMs from *Zfand5*^+/+^ and *Zfand5*^-/-^ mice (n=4). Statistical significance was assessed using Student’s *t*-test. *n.s.*, not significant. Data are presented as mean ± SD (error bars).

**Supplementary Fig. 3. Zfand5 has minimal effects on TLR2-mediated immune responses and signaling.**
BMDMs from *Zfand5*^+/+^ or *Zfand5*^-/-^ mice were stimulated with Pam_3_CSK_4_ (100 ng/ml) for the indicated times. (a) The mRNA expression levels of the indicated genes were analyzed by RT-qPCR. (b) Cytokine concentrations in culture supernatants were measured by ELISA. (c) Phosphorylation of IKKα/β, TBK1, IRF3, and MAPKs, as well as protein expression, was assessed by immunoblotting. Band intensities are presented as fold changes related to untreated controls after normalization to their unphosphorylated forms, and are shown below. Statistical significance was assessed using Student’s *t*-test. (**P* < 0.05, ***P* < 0.01, and ****P* < 0.001; *n.s.*, not significant). Data are representative of three independent experiments performed in triplicate, and are shown as mean ± SD (error bars).

**Supplementary Fig. 4 Zfand5 deficiency enhances TLR3/4 signaling and cytokine expression in BMDCs.**
(a) Bone marrow–derived dendritic cells (BMDCs) were generated from bone marrow cells isolated from *Zfand5*^+/+^ or *Zfand5*^-/-^ mice. Cells were collected and stained with antibodies against CD45, CD11c, B220, CD11b, XCR1, and SIRPα, followed by flow cytometric analysis. The differentiated BMDC population (CD45⁺CD11c⁺) exceeded 90% in both genotypes. (b-e) *Zfand5*^+/+^ and *Zfand5*^-/-^ BMDCs were stimulated with LPS (1 μg/ml) (b and d) or poly(I:C) (60 μg/ml) (c and e) for the indicated times. Cell lysates were collected and assessed by immunoblotting using the indicated antibodies (b and c). The mRNA expression of the indicated genes was analyzed by RT-qPCR (d and e). Band intensities are presented as fold changes related to untreated controls after normalization to their unphosphorylated forms, and are shown below. Statistical significance was assessed using Student’s *t*-test. (**P* < 0.05, ***P* < 0.01, and ****P* < 0.001; *n.s.*, not significant). Data are representative of two independent experiments performed in triplicate, and are shown as mean ± SD (error bars).

**Supplementary Fig. 5. Zfand6 has minimal impact on** **TLR4-mediated signaling and TRIF degradation.**
(a-c) sh*Luc*- and sh*Zfand6*-expressing RAW 264.7 cells were treated with LPS (100 ng/ml) for the indicated times. The mRNA expression of the indicated gene was analyzed by RT-qPCR (a, b). Phosphorylation of IKKα/β, TBK1, IRF3, and MAPKs, along with total protein levels in cell lysates was assessed by immunoblotting (c). Band intensities are presented as fold changes related to untreated controls after normalization to their unphosphorylated forms, and are shown below. (d, e) sh*Luc*- and sh*Zfand6*-expressing RAW 264.7 cells were pretreated with CHX (2 μg/ml) for 30 mins prior to LPS (100 ng/ml) stimulation for the indicated times. Cell lysates were analyzed by immunoblotting using the indicated antibodies. TRIF and MyD88 band intensities were normalized to GAPDH expression and compared with untreated controls. Quantification of three independent experiments is shown in the right panels. Statistical significance was determined using Student’s *t*-test (**P* < 0.05, ***P* < 0.01, ****P* < 0.001; *n.s.*, not significant). Data represent three independent experiments performed in triplicate and are presented as mean ± SD (error bars).

**Supplementary Fig. 6. Zfand5 regulates TRIF-dependent, but not MyD88-dependent, gene expression following TLR3/4 activation.**
(a) *Zfand5***^+/+^**, *Zfand5***^-/-^**, and *Zfand5***^-/-^***Trif***^-/-^** immortalized bone marrow-derived macrophages (iBMDMs) were analyzed for Zfand5 and TRIF protein expression by immunoblotting. (b) *Zfand5***^+/+^**, *Zfand5***^-/-^**, and *Zfand5***^-/-^***Trif***^-/-^** iBMDMs were stimulated with LPS (100 ng/ml) or poly(I:C) (30 μg/ml). At the indicated time points, total RNA was isolated, and the mRNA levels of the indicated genes were quantified by RT-qPCR. Statistical significance was determined using Student’s *t*-test (**P* < 0.05, ***P* < 0.01, ****P* < 0.001; *n.s.*, not significant). Data represent two independent experiments performed in triplicate and are presented as mean ± SD (error bars).

**Supplementary Fig. 7. Reconstitution of Zfand5 attenuates enhanced TLR4-driven immune responses in Zfand5-deficient RAW 264.7 cells.**
*Zfand5*^+/+^, *Zfand5*^-/-^, and *Zfand5*^-/-^ RAW 264.7 cells **reconstituted with Flag-tagged Zfand5** were stimulated with LPS (100 ng/ml) for the indicated time points. (a) Cell lysates were collected and subjected to **immunoblotting** using the indicated antibodies. (b) Total RNA was isolated, and the mRNA levels of the indicated genes were quantified by RT-qPCR. Statistical significance was determined using Student’s *t*-test (*P < 0.05, **P < 0.01, ***P < 0.001; n.s., not significant). Data **are representative of** two independent experiments performed in triplicate and are presented as mean ± SD (error bars).

**Supplementary Fig. 8. Both the A20 zinc-finger and CTR domains are required for Zfand5-mediated TRIF degradation and downstream signaling.**
*Zfand5*^-/-^ RAW 264.7 cells were reconstituted with a Tet-inducible vector expressing Flag-tagged wild-type or mutant Zfand5 constructs, as indicated. (a, b) Cells were pretreated with CHX (2 μg/ml) for 30 mins, followed by stimulation with LPS (100 ng/ml) plus CHX or poly(I:C) (100 μg/ml) plus CHX for the indicated times. Cell lysates were analyzed by immunoblotting with the indicated antibodies. TRIF band intensities were normalized to tubulin levels and compared to those of untreated cells. Quantification from three independent experiments is shown in the right panels. (c) Cells were treated with LPS (100 ng/ml) for the indicated times. Phosphorylation of IKKα/β, TBK1, IRF3, and the expression of other indicated proteins in cell lysates were assessed by immunoblotting. Band intensities are presented as fold changes relative to untreated controls after normalization to their unphosphorylated forms. Quantification of three independent experiments is shown in the right panels. Statistical analysis was calculated using Student’s *t*-test (**P* < 0.05, *n.s.*, not significant). Data are representative of three independent experiments and are presented as mean ± SD (error bars).

**Supplementary Table 1. Complete blood counts of *Zfand5*^+/+^ and *Zfand5*^-/-^ mice.**

| **Parameters** | **Unit** | ***Zfand5*^+/+^**  **(mean±SD)** | ***Zfand5*^-/-^**  **(mean±SD)** | ***p*-value** |
| --- | --- | --- | --- | --- |
| RBC | M/μL | 10.83±0.03 | 10.37±0.83 | 0.3898 |
| HGB | g/dL | 15.87±0.50 | 15.07±1.31 | 0.3780 |
| HCT | % | 55.70±1.57 | 53.77±5.72 | 0.6026 |
| MCV | fL | 51.50±1.30 | 51.73±1.42 | 0.8439 |
| MCH | Pg | 14.63±0.51 | 14.53±0.15 | 0.7625 |
| MCHC | g/dL | 28.43±0.25 | 28.07±0.64 | 0.4052 |
| RET | K/dL | 445.20±70.95 | 356.17±30.34 | 0.1163 |
| RET | % | 4.11±0.66 | 3.43±0.09 | 0.1540 |
| PLT | K/μL | 849.67±188.47 | 880.33±39.07 | 0.7963 |
| WBC | K/μL | 4.14±0.66 | 4.31±1.13 | 0.8913 |
| NEUT | K/μL | 1.19±1.57 | 0.97±1.13 | 0.8622 |
| LYMPH | K/μL | 2.85±1.05 | 3.21±1.22 | 0.7156 |
| MONO | K/μL | 0.06±0.03 | 0.07±0.02 | 0.7489 |
| EO | K/μL | 0.03±0.02 | 0.04±0.03 | 0.4918 |
| BASO | K/μL | 0.01±0.00 | 0.00±0.01 | 0.1161 |
| NEUT | % | 23.47±25.12 | 20.80±26.33 | 0.9051 |
| LYMPH | % | 73.87±24.04 | 76.53±25.75 | 0.9020 |
| MONO | % | 1.43±0.51 | 1.57±0.15 | 0.6885 |
| EO | % | 0.97±0.80 | 1.03±0.12 | 0.9113 |
| BASO | % | 0.27±0.12 | 0.07±0.12 | 0.1012 |

RBC: Red blood cells, HGB: Hemoglobin, HCT: Hematocrit, MCV: Mean corpuscular volume, MCH: Mean corpuscular Hemoglobin, MCHC: Mean corpuscular Hemoglobin concentration, RET: Reticulocyte, PLT: Platelet, WBC: White blood cells, NEUT: Neutrophils, LYMPH: Lymphocytes, MONO: Monocytes, EO: Eosinophils, BASO: Basophils.

**Supplementary Table 2. Primer sequences used for RT-qPCR analysis.**

| m*Cyclophilin* | Forward: 5’-ATGTGCCAGGGTGGTGACTTT-3’ |
| --- | --- |
|  | Reverse: 5’-TTGCCATCCAGCCATTCAGTC-3’ |
| m*Ifnb* | Forward: 5’-GAAGAGTTACACTGCCTTTGCC-3’ |
|  | Reverse: 5’-CAACAATAGTCTCATTCCACCC-3’ |
| m*Il1b* | Forward: 5’-GAACTCAACTGTGAAATGCCACC-3’ |
|  | Reverse: 5’-CCACAGCCACAATGAGTGATACT-3’ |
| m*Il6* | Forward: 5’-ACAAGAAAGACAAAGCCAGAGTC-3’ |
|  | Reverse: 5’-ATTGGAAATTGGGGTAGGAAG-3’ |
| m*Il10* | Forward: 5’-TGGGTTGCCAAGCCTTATCGG-3’ |
|  | Reverse: 5’-ACCTGCTCCACTGCCTTGCTC-3’ |
| m*Il12b* | Forward: 5’-AGACCCTGCCCATTGAACTG-3’ |
|  | Reverse: 5’-GAAGCTGGTGCTGTAGTTCTCATATT-3’ |
| m*Rantes* | Forward: 5’-GACACCACTCCCTGCTGCTTTG-3’ |
|  | Reverse: 5’-GATGTATTCTTGAACCCACTTCTT-3’ |
| m*Tnf* | Forward: 5’-GTCTACTGAACTTCGGGGTGATC-3’ |
|  | Reverse: 5’-TCCACTTGGTGGTTTGCTACG-3’ |
| m*Zfand5* | Forward: 5’-CCCAAACCAAAGAAGAACAGA-3’ |
|  | Reverse: 5’-GTAACGGTGAAGTCCACAAAA-3’ |
| m*Zfand6* | Forward: 5’-GCTAGCATGGCTCAAGAAACTAATCAC-3’ |
|  | Reverse: 5’-GGCGCGCCAAATCTTCTGGATCTTTTCAC-3’ |
| m*Cxcl2* | Forward: 5’- GCGTCACACTCAAGCTCTG-3’ |
|  | Reverse: 5’- CCAACCACCAGGCTACAGG-3’ |
